# The nucleocapsid protein of rice stripe virus in cell nuclei of vector insect regulates viral replication

**DOI:** 10.1007/s13238-021-00822-1

**Published:** 2021-03-06

**Authors:** Wan Zhao, Junjie Zhu, Hong Lu, Jiaming Zhu, Fei Jiang, Wei Wang, Lan Luo, Le Kang, Feng Cui

**Affiliations:** 1 State Key Laboratory of Integrated Management of Pest Insects and Rodents, Institute of Zoology, Chinese Academy of Sciences, Beijing, 100101 China; 2 CAS Center for Excellence in Biotic Interactions, University of Chinese Academy of Sciences, Beijing, 100049 China

**Keywords:** rice stripe virus, nucleocapsid protein, nuclear localization, importin α, YY1

## Abstract

Rice stripe virus (RSV) transmitted by the small brown planthopper causes severe rice yield losses in Asian countries. Although viral nuclear entry promotes viral replication in host cells, whether this phenomenon occurs in vector cells remains unknown. Therefore, in this study, we systematically evaluated the presence and roles of RSV in the nuclei of vector insect cells. We observed that the nucleocapsid protein (NP) and viral genomic RNAs were partially transported into vector cell nuclei by utilizing the importin α nuclear transport system. When blocking NP nuclear localization, cytoplasmic RSV accumulation significantly increased. In the vector cell nuclei, NP bound the transcription factor YY1 and affected its positive regulation to *FAIM*. Subsequently, decreased *FAIM* expression triggered an antiviral caspase-dependent apoptotic reaction. Our results reveal that viral nuclear entry induces completely different immune effects in vector and host cells, providing new insights into the balance between viral load and the immunity pressure in vector insects.

**Supplementary Information:**

The online version of this article (10.1007/s13238-021-00822-1) contains supplementary material, which is available to authorized users.

## Introduction

As a primary grain crop, rice feeds more than half of the world’s population (Sasaki and Burr, 2000). Rice stripe virus (RSV), a single-stranded RNA virus of the genus *Tenuivirus*, causes one of the most destructive rice diseases and up to an 80% incidence with 30% to 40% yield losses in the rice fields of Asian countries (Wei et al., 2009; Zhou et al., 2012). The genome of RSV consists of four RNA segments. RNA1 is negative-sense and encodes RNA-dependent RNA polymerase (RdRp). The other three segments are ambisense and each encodes two proteins, i.e., NS2 and NSvc2 encoded by RNA2, NS3 and nucleocapsid protein (NP) by RNA3, and SP and NSvc4 by RNA4 (Toriyama, 1986; Hamamatsu et al., 1993). RSV is a typical persistent-propagative phytovirus that is efficiently transmitted by the small brown planthopper *Laodelphax striatellus*. Although it replicates and assembles in the cytoplasm of both planthopper and rice cells, RSV performs differently when living in the two organisms, exhibiting different pathogenicities (Zhao et al., 2016a), replication and gene expression levels (Zhao et al., 2019), genomic 3′-termini (Zhao et al., 2018), and virus-derived siRNAs (Yang et al., 2018).

Nuclear entry of viruses or viral proteins has been demonstrated to be closely related to viral replication, assembly and pathogenicity in host cells (Hiscox, 2007; Audsley et al., 2016). As for nucleorhabdoviruses, which replicate in the nuclei of the infected cells, it is understandable that viral proteins and particles accumulate in the nuclei (Richardson and Sylvester, 1968). For the RNA viruses that replicate in the cytoplasm, structural or nonstructural proteins of these viruses can also enter the nuclei of host cells, such as human flavivirus (Byk and Gamarnik, 2016), hepacivirus (Falcón et al., 2003), coronavirus (Hiscox et al., 2001), arterivirus (Rowland et al., 1999) and alphavirus (Michel et al., 1990), and plant potyviruses (Ivanov et al., 2014) and umbraviruses (Kim et al., 2007). To cross the nuclear membrane, viruses typically utilize the host classical importin (karyopherin) α/β nuclear transport pathway (Hiscox, 2007). Viral nuclear entry has been demonstrated to be beneficial for viral proliferation by inhibiting host antiviral responses, such as the apoptosis or interferon signaling pathways (Marusawa et al., 1999; Ashour et al., 2009). Similarly, nuclear entry of insect-vectored phytoviruses and arboviruses has also been found to promote viral replication in host cells (Liu et al., 2007; Xiong et al., 2009; Zheng et al., 2017). However, little is known as to whether these cytoplasmic RNA viruses or viral proteins can enter the nuclei of vector insect cells and have important effects on viral replication in these cells.

The replication of RSV in planthoppers is under the elaborate control of the immune system. We previously showed that the overall immune reaction toward RSV is much stronger in the salivary gland than in the gut (Zhao et al., 2016b). RSV activates the c-Jun N-terminal kinase (JNK) signaling pathway to promote viral replication in planthoppers (Wang et al., 2017). However, RSV significantly upregulates the expression of an angiotensin-converting enzyme, which has negative effects on RSV *SP* expression (Wang et al., 2019). The results of our recent study has provided basic clues regarding the interaction between the NP of RSV and an importin α protein of planthoppers (Zhu et al., 2020), suggesting that RSV or at least its NP can enter vector nuclei through the importin α/β pathway to influence immune reactions.

In this study, we assessed the occurrence of the nuclear entry of RSV and the potential impacts on viral performance in the vector insect. We observed, for the first time, that the NP and viral genomic RNAs of RSV are able to enter the nuclei of vector insects. The nuclear entry of NP occurs by utilizing the importin α nuclear transport system of vector cells. Unlike RNA viruses in host cells, the nuclear localization of RSV triggers an antiviral apoptotic response to control viral replication levels in vector cells. Thus, the results of our study provide new insights into the balance between viral load and the immunity pressure in vector insects.

## Results

### RSV ribonucleoprotein particles localize in cell nucleus of planthoppers

**Figure 1:**
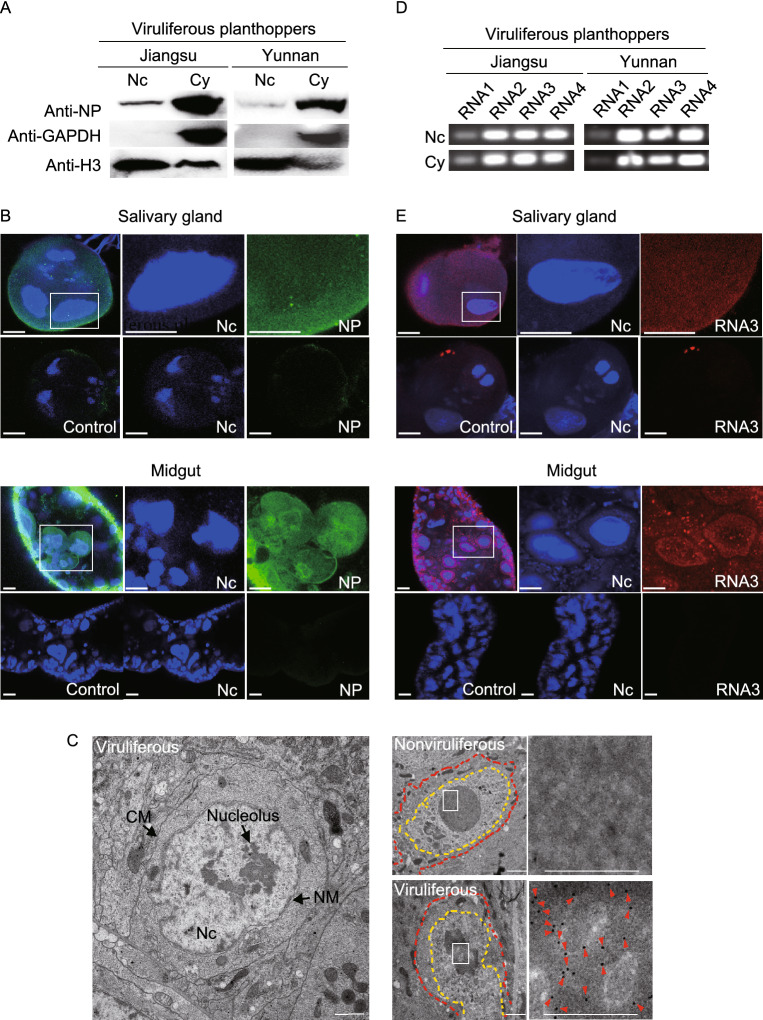
**RSV ribonucleoprotein particles localize in the nuclei of planthopper cells**. (A) Western blot results show the nuclear location of nucleocapsid protein (NP) in the nuclear (Nc) and cytoplasmic (Cy) extracts from viruliferous planthoppers bearing Jiangsu or Yunnan RSV isolates using a monoclonal anti-NP antibody. Reference proteins for nuclear and cytoplasmic proteins were histone H3 and GAPDH, respectively, which were observed using anti-H3 and anti-GAPDH monoclonal antibodies, respectively. (B) Immunohistochemistry showing the NP nuclear location in the salivary gland and midgut cells. The green signal is from an Alexa Fluor 488-labeled anti-NP monoclonal antibody. The blue signal is the nucleus (Nc) stained with Hoechst. The boxed region is enlarged and shown in two different panels on the right side. The samples without the treatment of anti-NP antibody are shown as negative controls. Scale bars: 10 μm. (C) Colloidal gold immunoelectron microscopy showing the NP nuclear location in the midgut cells. The left panel is the viruliferous midgut section without treatment of a monoclonal anti-NP antibody. Nc, nucleus; NM, nuclear membrane; CM, cell membrane. In the right panels, the cells and nuclei are outlined by red-dotted and yellow-dotted lines, respectively. NP was labeled with 10 nm-gold particles (red strangles). The boxed region is enlarged and shown in the panel on the right side. Scale bars: 1 μm. (D) RSV genomic RNA segments were amplified from nuclear (Nc) and cytoplasmic (Cy) extracts from viruliferous planthoppers bearing the Jiangsu or Yunnan RSV isolates by reverse transcription-PCR. (E) *In situ* fluorescence hybridization to assess the viral genomic RNA3 location in the salivary gland and midgut cells. The red signal is from the digoxigenin (DIG)-labeled RNA3 probe, and the blue signal is the nucleus (Nc) stained with Hoechst. The boxed region is enlarged and shown in two different panels on the right side. The samples without the treatment of RNA3 probe are shown as negative controls. Scale bars: 10 μm

NP is RSV’s structural protein, localizing to the exterior of the viral particle and encapsidating each viral genomic RNA molecule to form ribonucleoprotein particles (RNPs), the minimal infectious unit (Toriyama, 1986). To assess the subcellular localization of viral NP in vector cells, we first separated the nuclear and cytoplasmic proteins from whole bodies of viruliferous planthoppers bearing Jiangsu or Yunnan RSV isolates. Western blot results showed that NP was present in vector cell nuclei and cytoplasm for both viral isolates (Fig. 1A). The nuclear localization of NP was further visualized in the salivary gland and midgut cells via immunohistochemistry assays (Fig. 1B). In addition, colloidal gold immunoelectron microscopy showed that many NP-conjugated gold particles accumulated in nuclei of the midgut cells, especially in the nucleolus (Fig. 1C). Because viral NP encapsidates genomic RNAs to form RNPs, we assessed whether viral genomic RNAs were also present in the nuclei. As expected, all four RNA segments of RSV were detected in both the nuclear and cytoplasmic extracts of viruliferous planthoppers bearing the Jiangsu or Yunnan RSV isolates by PCR amplification (Fig. 1D). Moreover, using a DIG-labeled RNA3 fragment as a probe, we observed that RNA3 was present in both the nuclei and cytoplasm of the salivary gland and midgut cells via *in situ* fluorescence hybridization (Fig. 1E). These results show that the RNPs of RSV are able to enter the cell nuclei of planthoppers.

### RSV NP enters cell nuclei of vector insects through interaction with importin α proteins

**Figure 2:**
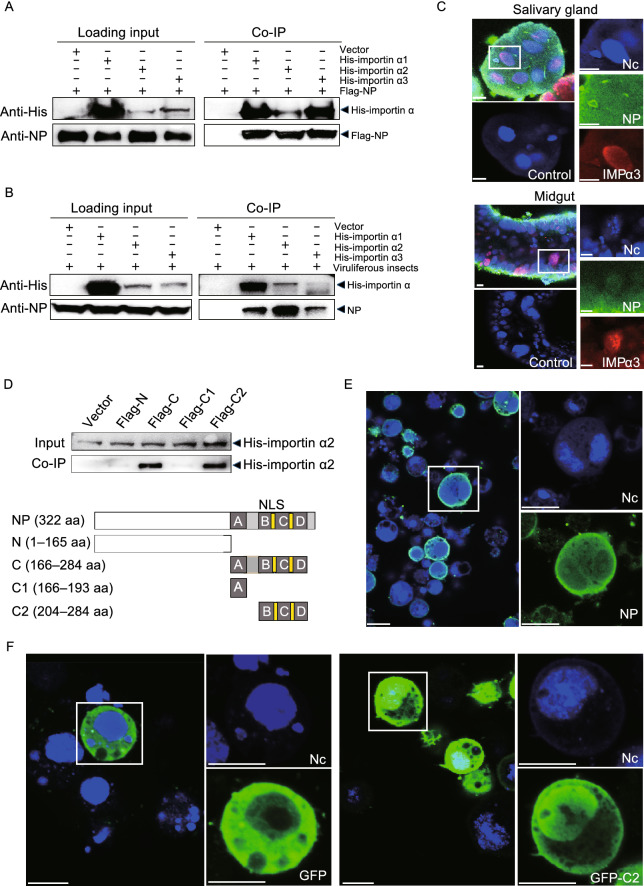
**RSV nucleocapsid protein interacts with importin α proteins via.** **the nuclear localization signal.** (A) Recombinantly expressed RSV nucleocapsid protein (NP) with a Flag-tag binds three recombinantly expressed His-importin α proteins in the co-immunoprecipitation (Co-IP) and Western blot assay. The expression products from the pET28a vector were used as a negative control. (B) Three recombinantly expressed His-importin α proteins pulled down the NP from viruliferous planthoppers in the Co-IP and Western blot assay. The expression products from the pET28a vector were used as a negative control. (C) Colocalization of importin α3 and NP examined in the salivary gland and midgut cells via immunohistochemistry analysis. The green signal is from an Alexa Fluor 488-labeled anti-NP monoclonal antibody. The red signal is from an Alexa Fluor 594-labeled anti-importin α3 (IMPα3) polyclonal antibody. The boxed region is enlarged and shown in three different panels on the right side. The samples without the treatment of primary antibodies are shown as negative controls. (D) Co-IP and Western blot assay for the interaction between the recombinantly expressed Flag-N, Flag-C, Flag-C1, or Flag-C2 fragments of NP and His-importin α2. Motifs A, B, C and D comprise the putative nuclear localization signals (NLS). The overlapping amino acid residues shared by the motifs are marked in yellow. The expression products from the pET28a vector were used as a negative control. (E) Immunohistochemistry analysis of the subcellular location of expressed NP in S2 cells. The green signal is from an Alexa Fluor 488-labeled anti-NP monoclonal antibody. The boxed region is enlarged and shown in two different panels on the right side. (F) Subcellular location of expressed GFP and the GFP-C2 fragment in S2 cells was observed. The boxed region is enlarged and shown in two different panels on the right side. Scale bars in 2C, 2E, and 2F: 10 μm. The blue signal is the nuclei (Nc) stained with Hoechst

Because the NP of RSV interacts with one importin α protein (i.e., importin α2 as designated below) of planthoppers (Zhu et al., 2020), and since viruses typically utilize the host importin α/β nuclear transport pathway (Hiscox, 2007), the importin α/β pathway may be involved in RSV nuclear entry. To test this hypothesis, we first assessed the interactions between the RSV NP and importin α proteins of planthoppers. There are three importin α genes encoded in the genome of the small brown planthopper (Zhu et al., 2017). Based on the sequence identities of these genes to *Drosophila* homologs, we named them importin α1 (GenBank registration number MT036051), importin α2 (MT036050), and importin α3 (MT036052) (Fig. S1A), which have predicted protein molecular weights of 57.9, 57.5, and 56.8 kDa, respectively. After recombinantly expressing these proteins with His-tags, all the three His-tagged importin α proteins were able to bind recombinantly expressed Flag-NP (Fig. 2A) and the NP from viruliferous planthoppers (Fig. 2B) in co-immunoprecipitation (Co-IP) assays. Thus, viral NP can interact with the three importin α proteins of the assayed planthoppers.

The colocalization of importin α proteins and NP was also examined in the salivary gland and midgut cells via immunohistochemistry assays. Three polyclonal antibodies against importin α1, α2 and α3 were generated. The anti-importin α2 and α3 antibodies recognized antigens expressed both *in vitro* and *in vivo* despite each antibody not being specific enough to recognize their unique importin α, while the anti-importin α1 antibody failed to detect importin α1 *in vivo* (Fig. S1B and S1C). The immunohistochemistry results obtained using the anti-importin α3 antibody confirmed that importin α3, or perhaps also importin α2, was primarily present in the nuclei of salivary gland and midgut cells and that NP and importin α proteins colocalized in the nuclei (Fig. 2C).

The ability of NP to enter the nucleus requires a nuclear localization signal (NLS). The predicted NLS in NP includes four motifs [166–193 aa (motif A), 204–234 aa (motif B), 232–260 aa (motif C), and 255–284 aa (motif D)], although they have low confidence scores (<5). To elucidate whether these putative NLS motifs of NP take part in binding with importin α proteins, we recombinantly expressed four NP fragments [from 1 to 165 aa (N), 166 to 284 aa (C, including the four motifs), 166 to 193 aa (C1, motif A), and 204 to 284 aa (C2, including motifs B, C, and D)] with a Flag tag and tested their interactions with His-importin α2, which has been found to interact with the intact NP (Zhu et al., 2020). The results showed that fragments C and C2 pulled down His-importin α2, whereas fragments N and C1 did not (Fig. 2D), suggesting that motifs B, C, and D take part in binding to importin α proteins. Next, we tested whether motifs B, C, and D have a nuclear localization function through the recombinant expression of fragment C2 with GFP in *Drosophila* S2 cells. Intact NP was observed in both the nuclei and cytoplasm of S2 cells in immunohistochemistry assays (Fig. 2E), demonstrating that the NLS of NP functioned in S2 cells. More GFP-C2 proteins were located in the nuclei than the cytoplasm in 56.7% (34 out of 60) S2 cells, while more GFP proteins were observed in the cytoplasm than the nuclei in 88.8% (71 out of 80) cells (Fig. 2F). Thus, motifs B, C, and D harbor the NLS of NP, mediating the ability of NP to enter cell nuclei and the interaction of NP with importin α proteins.

### Inhibition of NP nuclear entry significantly promotes RSV replication

**Figure 3:**
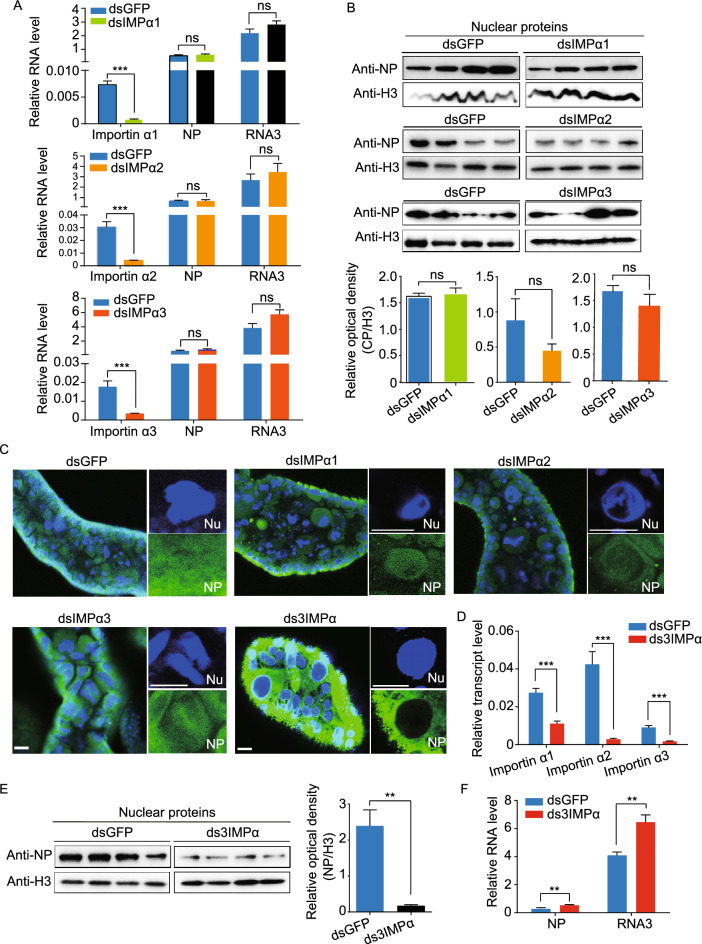
**Inhibition of NP nuclear entry significantly promotes RSV accumulation in planthoppers**. (A) Relative RNA levels of RSV *NP* and genomic RNA3 7 d after the injection of dsRNA of *importin α1* (dsIMPα1), *importin α2* (dsIMPα2), *importin α3* (dsIMPα3), or *GFP* (dsGFP), as measured by quantitative real-time PCR (qPCR). The RNA levels of *NP* and RNA3, and the transcript levels of *importin α* genes are normalized to that of *EF2*. (B) The nuclear accumulation of NP in the nuclear protein extracts of viruliferous planthoppers after the injection of dsIMPα1, dsIMPα2, dsIMPα3 or dsGFP for 7 d, as assessed by Western blot assay. NP was detected using a monoclonal anti-NP antibody. The reference protein for the nuclear proteins was histone H3, which was detected using a monoclonal anti-H3 antibody. Four independent replicates are shown for each group. The relative optical densities of NP to that of H3 were calculated. (C) Immunohistochemistry analysis of the subcellular location of NP in the midgut cells after the injection of dsIMPα1, dsIMPα2, dsIMPα3, a mixture of dsRNA of the three *importin α* genes (ds3IMPα), or dsGFP for 7 d. NP was detected using a monoclonal anti-NP antibody. The blue signal is the nuclei (Nc) stained with Hoechst. The boxed region is enlarged and shown in two different panels on the right side. Scale bars: 20 μm. (D) The relative transcript levels of *importin α1*, *α2* and *α3* after the injection of ds3IMPα or dsGFP for 7 d measured by qPCR. (E) Nuclear accumulation of NP in the nuclear protein extracts of viruliferous planthoppers after the injection of ds3IMPα or dsGFP for 7 d, as assessed via Western blot assay. Four independent replicates are shown for each group. The relative optical densities of NP to that of H3 are calculated. (F) Relative RNA levels of RSV *NP* and genomic RNA3 after injection of ds3IMPα or dsGFP for 7 d measured by qPCR. ns, no significant difference. **P < 0.01. ***P < 0.001

To assess the impact of NP nuclear localization on RSV replication, we injected double-stranded RNA (dsRNA) of *importin α1*, *α2*, or *α3* into viruliferous planthoppers. After the administration of the dsRNAs, the transcript levels of the three genes decreased more than 85% at 7 d post injection (dpi) (Fig. 3A). However, the amount of NP in the nuclear proteins isolated from whole bodies of insects was not significantly affected by the knockdown of any of *importin α* gene as assessed by Western blot assays (Fig. 3B). In addition, we also examined the situation in the midgut, which is the main tissue for RSV replication. No significant reduction of NP in the nuclei of midgut cells was observed *in situ* through immunohistochemistry assays, although a slight reduction was observed in the ds*importin α2*-RNA or ds*importin α3*-RNA injection groups (Fig. 3C). Probably due to functional complementarity of the three importin α proteins, interfering with the expression of one *importin α* gene did not affect the entrance of NP to nuclei. Consequently, the viral load in terms of the RNA levels of *NP* and genomic RNA3 was not altered in planthoppers when one of the three *importin α* genes was knocked down (Fig. 3A).

Subsequently, we knocked down all the three *importin α* genes in viruliferous planthoppers by injecting a mixture of dsRNAs for the three *importin α* genes. Interreference efficiencies of 82%, 92%, and 64% were obtained for the *importin α1*, *α2*, and *α3* genes, respectively (Fig. 3D). Compared to the insects injected with ds*GFP*-RNA, the level of NP protein in the nuclei of insects injected with the ds*importin α*-RNA mixture was decreased by 89% (Fig. 3E). The immunohistochemistry assay results observed using midgut cells showed that little NP was present in the nuclei, while more NP accumulated in the cytoplasm after knockdown of the three *importin α* genes (Fig. 3C). Furthermore, the RNA levels of *NP* and genomic RNA3 significantly increased in the whole bodies (Fig. 3F). Therefore, interference with *importin α* gene expression significantly retarded NP nuclear entry and promoted RSV replication in planthoppers.

### Binding of NP to the transcription factor Yin Yang-1 in nuclei suppresses RSV replication

The cause for the inhibitory effect of NP nuclear entry on RSV replication was further evaluated by screening for nuclear proteins that interact with NP through Co-IP assays followed by mass spectrometry identification. Sixty-one NP-interacting nuclear proteins with a peptide score higher than 80 were identified. These proteins included 30 ribosomal proteins, 12 ATP-related proteins, nine cytoskeleton-related proteins, four heat shock proteins, four oxidases, the transcription factor Yin Yang-1 (YY1), and the histone H4 (Table S1). Only YY1 and histone H4 primarily function in the nucleus, while the other candidate proteins exhibit nucleocytoplasmic shuttling that primarily have roles in the cytoplasm. Because YY1 is a ubiquitous transcription factor that takes part in mammalian immune reactions by regulating the transcription of immune-related genes (Austen et al., 1997; Gordon et al., 2006), YY1 was selected as a key NP-interacting candidate for further studies.

**Figure 4:**
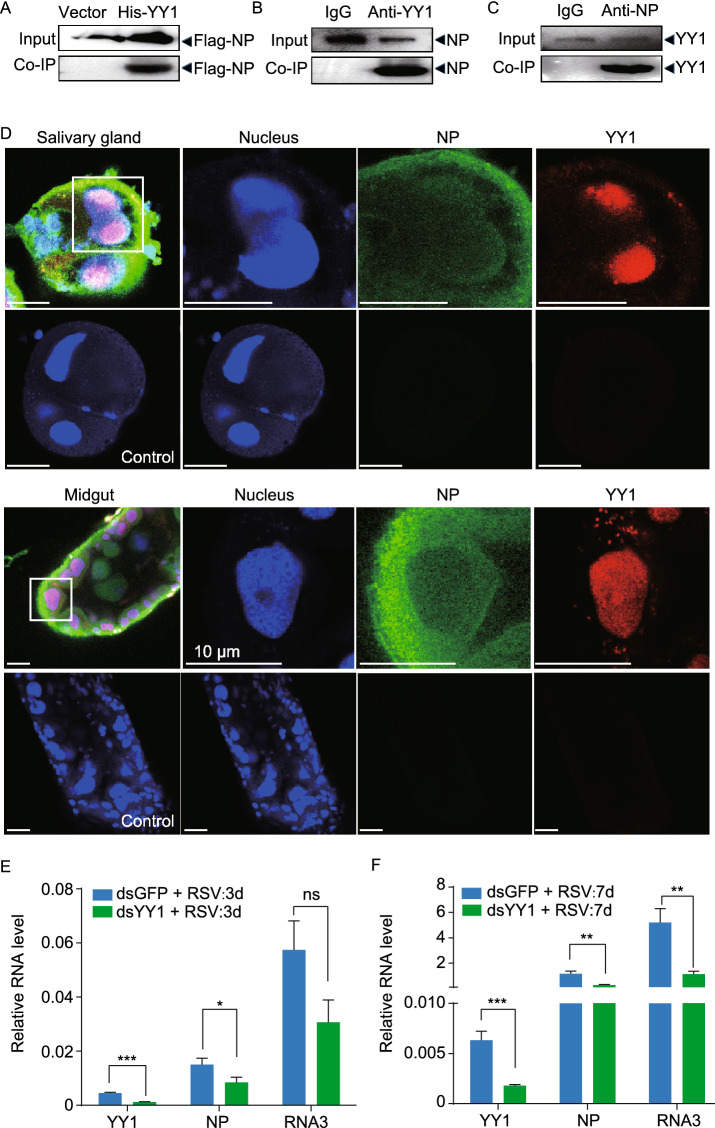
**Binding of NP to YY1 in the nuclei leads to an antiviral reaction in planthoppers**. (A) Recombinantly expressed His-YY1 binds recombinantly expressed Flag-NP in the co-immunoprecipitation (Co-IP) and Western blot assay. The expression products from the pET28a vector were used as a negative control. (B) NP was pulled down from viruliferous planthoppers in the Co-IP and Western blot assay using an anti-human YY1 polyclonal antibody. An IgG antibody was used as a negative control. (C) YY1 was pulled down from viruliferous planthoppers in the Co-IP and Western blot assay using an anti-NP monoclonal antibody. An IgG antibody was used as a negative control. (D) Subcellular colocalization of NP and YY1 in the salivary gland and midgut cells of viruliferous planthoppers as determined by immunohistochemistry analysis. The green signal is from an Alexa Fluor 488-labeled anti-NP monoclonal antibody. The red signal is from an Alexa Fluor 594-labeled anti-human YY1 polyclonal antibody. The blue signal is the nuclei stained with Hoechst. The boxed region is enlarged and shown in three different panels on the right side. The samples without the treatment of primary antibodies are shown as negative controls. Scale bars: 20 μm. (E) Relative RNA levels of RSV *NP* and genomic RNA3 and *YY1* in the planthoppers at 3 d after injection of dsRNA of *YY1* (dsYY1) and RSV crude preparations measured by quantitative real-time PCR (qPCR). The control group was injected with dsRNA of *GFP* (dsGFP) and RSV crude preparations. The RNA levels of *NP*, RNA3, and *YY1* are normalized to that of *EF2*. (F) The relative RNA levels of RSV *NP* and genomic RNA3 and *YY1* in the planthoppers at 7 d after injection of dsRNA of *YY1* (dsYY1) and RSV crude preparations measured by qPCR. ns, no significant difference. *P < 0.05. **P < 0.01. ***P < 0.001

The interaction between YY1 and NP was confirmed by multiple lines of evidence. The *YY1* gene (GenBank registration number MT113358) of planthoppers putatively encodes a 41.7 kDa protein. After being recombinantly expressed with a His-tag, His-YY1 pulled down the Flag-NP *in vitro* (Fig. 4A). The protein sequence of planthopper YY1 showed a 67.1% identity to the human YY1, and a 74.4% identity to the region between amino acid residues 157 and 384 of human YY1, based on which the anti-human YY1 polyclonal antibody is generated (Fig. S2). Using this anti-human YY1 polyclonal antibody NP was pulled down from viruliferous planthoppers in a Co-IP assay (Fig. 4B). Similarly, YY1 was pulled down from viruliferous planthoppers using the anti-NP monoclonal antibody (Fig. 4C). In addition, the colocalization of YY1 and NP in the nuclei of salivary gland and midgut cells was observed in the immunohistochemistry staining results (Fig. 4D).

To determine whether YY1 participates in immune regulation of RSV replication, we simultaneously injected ds*YY1*-RNA and insect-derived RSV crude preparations into nonviruliferous planthoppers. This insect-derived RSV can successfully circulate and replicate in planthoppers through microinjection (Chen et al., 2020). The expression of YY1 was inhibited at 3 and 7 dpi (Fig. 4E and 4F). Compared to that observed in the ds*GFP*-RNA injection group, the RNA levels of *NP* were significantly downregulated at 3 and 7 dpi, while the viral genomic RNA3 levels were downregulated at 7 dpi (Fig. 4E and 4F). Thus, *YY1* knockdown reduced RSV replication in planthoppers, consistent with the inhibitory effect of NP nuclear entry on RSV replication. Binding of NP with YY1 in the nuclei could inhibit the original functions of YY1 to reduce RSV replication.

### YY1 positively regulates the expression of *Fas apoptotic inhibitory molecule* to modulate cell apoptosis

The downstream immune-related genes regulated by YY1 were searched for by comparing the consensus binding motif of YY1 (5′-CCGCCATNTT-3′) (Kim and Kim, 2009) with the promoter regions in the planthoppers. One hundred nine genes were predicted to contain the YY1 binding motif in their promoter regions (Table S2). Only six genes were involved in the antiviral response, including a *cytochrome P450* (*P450*), *Fas apoptotic inhibitory molecule* (*FAIM*), *ubiquitin carrier protein E2* (*E2*), *ubiquitin carboxyl-terminal hydrolase 8* (*UCTH8*), *putative E3 ubiquitin-protein ligase HERC2* (*HERC2*), and *peroxisomal targeting signal 1 receptor* (*PTSR*) (Bień et al., 2015; Davis and Gack, 2015; Dixit et al., 2010; Walubo, 2007).

**Figure 5:**
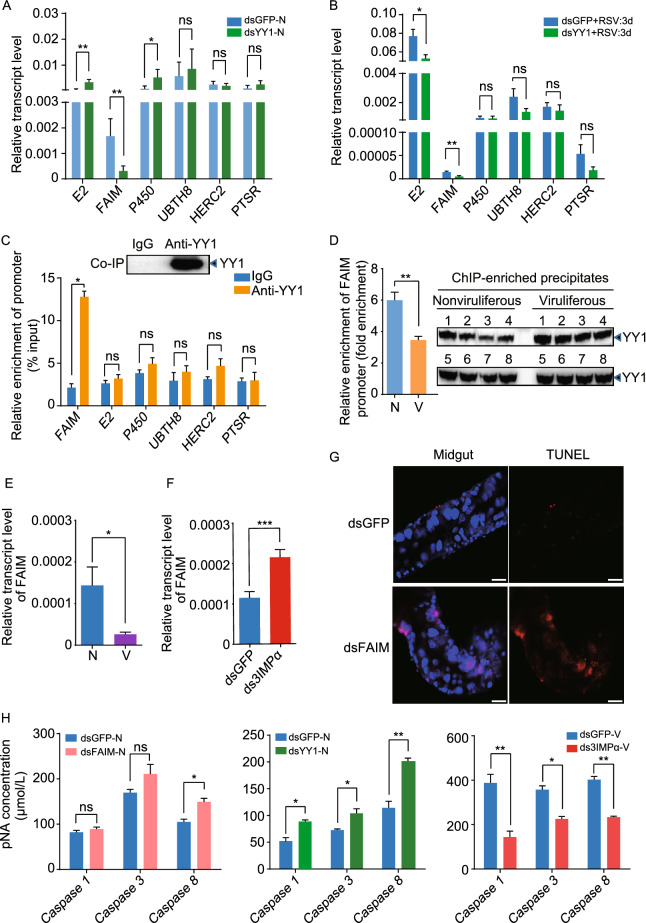
**YY1 positively regulates the expression of** ***FAIM*** **to modulate cell apoptosis**. (A) Relative transcript levels of the potential target genes of YY1 in nonviruliferous planthoppers (N) at 7 d after injection of dsRNA of *YY1* (dsYY1) or *GFP* (dsGFP) measured by quantitative real-time PCR (qPCR). The transcript levels of these genes were normalized to that of *EF2*. (B) The relative transcript levels of the potential target genes of YY1 in the planthoppers at 3 d after injection of dsYY1 and RSV crude preparations measured by qPCR. The control group was injected with dsGFP and RSV crude preparations. (C) Relative enrichment of promoter regions of the putative targets of YY1 measured by chromatin immunoprecipitation (ChIP) combined with qPCR. The anti-human YY1 polyclonal antibody efficiently captured the YY1 from nonviruliferous planthoppers in the ChIP as shown by the Western blot. IgG was used instead of the anti-YY1 antibody in the immunoprecipitation step as the negative control. (D) Relative enrichment of *FAIM* promoter binding with YY1 to that of IgG immunoprecipitation group (fold enrichment) in viruliferous (V) and nonviruliferous (N) planthoppers measured by ChIP-qPCR. The comparable amounts of YY1 precipitated from viruliferous and nonviruliferous insects were quantified using the Western blot assay with the anti-human YY1 polyclonal antibody. Eight biological replicates were prepared for each group. (E) The relative transcript levels of *FAIM* to that of *EF2* in nonviruliferous and viruliferous planthoppers. N, nonviruliferous insects. V, viruliferous insects. (F) The relative transcript levels of *FAIM* to that of *EF2* in viruliferous insects at 7 d after injection of dsRNA mixture of the three *importin α* genes (ds3IMPα) or *GFP* (dsGFP). (G) Apoptotic response in the midgut cells of nonviruliferous planthoppers at 7 d after injection of dsRNA of *FAIM* (dsFAIM) or dsGFP assayed with the TUNEL assay. Scale bars: 20 μm. (H) Caspase activities in planthoppers measured with the human caspase 1, 3 and 8 Activity Assay kits. The activities were calculated as µmol/L of the product p-nitroaniline (pNA). For nonviruliferous planthoppers (N), dsFAIM or dsYY1 was injected. For viruliferous planthoppers (V), ds3IMPα was injected. The control group was injected with dsGFP. The caspase activities were measured at 7 d after injection. ns, no significant difference. *P < 0.05. **P < 0.01. ***P < 0.001

To determine whether the six genes are targets of YY1, we measured their transcript levels after injecting insects with ds*YY1*-RNA. In the nonviruliferous insects, the transcript level of *FAIM* was downregulated, while that of *E2* and *P450* was upregulated after knockdown of YY1 at 7 d (Figs. 5A and S3A). In the planthoppers bearing RSV for 3 d, the transcript levels of *FAIM* and *E2* were downregulated, whereas those of the other four genes were unchanged after knockdown of YY1 (Figs. 5B and 4D). Only *FAIM* showed a consistent transcriptional decrease concomitant with the reduction in YY1 levels. In parallel, we performed chromatin immunoprecipitation (ChIP) assays combined with quantitative real-time PCR (qPCR) to directly capture and quantify the DNA levels of the promoter regions of the six genes. The anti-human YY1 polyclonal antibody efficiently captured the YY1 from the planthoppers in the ChIP assay, and a significantly higher amount of the *FAIM* promoter sequence was pulled down with the anti-YY1 antibody compared to that observed using IgG, while the promoter sequences of the other five genes were not enriched (Fig. 5C).

To explore whether and how RSV NP interferes with the transcription function of YY1, we applied ChIP-qPCR to compare the amount of *FAIM* promoter DNA that bound with YY1 from viruliferous and nonviruliferous planthoppers. Equal amounts of YY1 proteins immunoprecipitated from viruliferous and nonviruliferous planthoppers were ensured using ELISA and Western blot assay. The DNA contained in the YY1 was extracted and the *FAIM* promoter was quantified with qPCR. The results showed that the amount of *FAIM* promoter DNA bound with YY1 was much lower in the viruliferous planthoppers than in the nonviruliferous planthoppers (Fig. 5D). This result indicated that RSV NP interfered with the binding of YY1 to the *FAIM* promoter. Furthermore, the transcription of *FAIM* was suppressed in viruliferous insects compared to nonviruliferous insects (Fig. 5E) and was activated in viruliferous insects when the expression of the three *importin α* genes were interfered (Figs. 5F and S3B). Therefore, the transcription factor YY1 positively regulates the expression of *FAIM*, which is affected by the nuclear entry of RSV NP.

Because FAIMs function as apoptosis inhibitors to protect against death receptor-triggered apoptosis in mammals (Segura et al., 2007), we speculated that FAIM has a similar role in planthoppers. The planthopper has only one FAIM (gene set number evm.model.Contig137.40), which is more similar in amino acid sequence to human FAIM1 (70.6% identity) than to FAIM2 (45.8% identity) or FAIM3 (38% identity). To assess the function of FAIM as an apoptosis inhibitor in planthoppers, we tested the effect of FAIM on apoptosis in midgut cells and whole bodies. When the expression of *FAIM* was knocked down in nonviruliferous insects via ds*FAIM*-RNA injection, an apoptotic response was induced, as more TUNEL-positive signals were observed in midgut cells in the TUNEL assay (Figs. 5G and S3C).

We identified and annotated four caspase genes (caspase-Nc, caspase-8, caspase-1a, and caspase-1c) corresponding to the homologs of the brown planthopper (*Nilaparvata lugens*) (Fig. S4). Although the caspases of the planthoppers did not cluster well with human caspases (Fig. S4), the caspase activities of the planthoppers could be measured using the kits used for human caspase 1, 3, and 8. Standard curves for the three caspases were generated with correlation coefficient (R^2^) values greater than 0.99 (Fig. S5). When the expression of *FAIM* or *YY1* was knocked down in nonviruliferous insects (Fig. S3A and S3C), the caspase activities in the whole bodies increased (Fig. 5H). In contrast, activation of *FAIM* by interfering with the expression of the three *importin α* genes in viruliferous insects significantly reduced the caspase activities (Figs. 5H and S3B). These results confirmed the role of FAIM in negatively regulating cell apoptosis in planthoppers.

### NP nuclear entry activates cell apoptosis to inhibit viral replication in planthoppers

**Figure 6:**
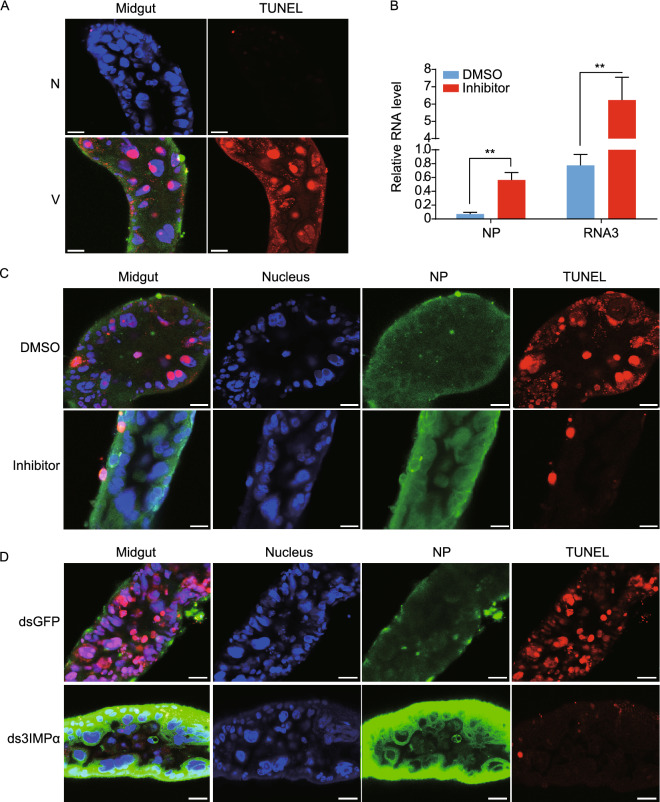
**NP nuclear entry activates cell apoptosis to inhibit viral replication in planthoppers.** (A) Apoptotic response in the midgut cells of nonviruliferous and viruliferous planthoppers as assessed by immunohistochemistry assays. N, nonviruliferous insects. V, viruliferous insects. The green signal is from an Alexa Fluor 488-labeled anti-NP monoclonal antibody. The red signal is from TUNEL label. The blue signal is the nuclei stained with Hoechst. (B) The relative RNA levels of RSV *NP* and genomic RNA3 in the midguts of viruliferous planthoppers at 6 d after injection of the pan-caspase inhibitor measured by quantitative real-time PCR. DMSO was injected in the control group. The RNA levels of *NP* and RNA3 are normalized to that of *EF2*. **P < 0.01. (C) Immunohistochemistry for the midguts of viruliferous planthoppers at 6 d after injection of the pan-caspase inhibitor. DMSO was injected in the control group. (D) Immunohistochemistry for the midguts of viruliferous planthoppers at 7 d after injection of dsRNA mixture of the three *importin α* genes (ds3IMPα). dsRNA of *GFP* (dsGFP) was injected in the control group. Scale bars in 6A, 6C, and 6D: 20 μm

The apoptotic status was compared between the midgut cells of viruliferous and nonviruliferous planthoppers. The TUNEL assay results showed that TUNEL-positive signals were detected in the midgut cells of viruliferous insects. In contrast, few TUNEL-positive cells were observed in the nonviruliferous insects (Fig. 6A), indicating that RSV increased the apoptotic response in the midgut, which is the primary organ for RSV replication.

To investigate the effect of cell apoptosis on RSV replication, we injected the pan-caspase inhibitor Z-VAD-FMK into viruliferous planthoppers. The RNA levels of *NP* and genomic RNA3 in the midgut significantly increased at 6 dpi compared to that observed in the DMSO-injected control group (Fig. 6B). The TUNEL assay results also showed that the apoptosis signals were greatly suppressed, and more NP accumulated in the midgut cells with the pan-caspase inhibitor treatment (Fig. 6C). When the expression of the three *importin α* genes was interfered, the TUNEL-positive signals in the midgut cells were dramatically reduced (Figs. 6D and S2B), corresponding to the significantly reduced caspase activities (Fig. 5G). In addition, more NP accumulated in the cytoplasm of cells compared to that observed in the control group (Figs. 3C and 6D). Therefore, NP nuclear entry induced cell apoptosis, which was detrimental to RSV replication in vector insects.

## Discussion

**Figure 7:**
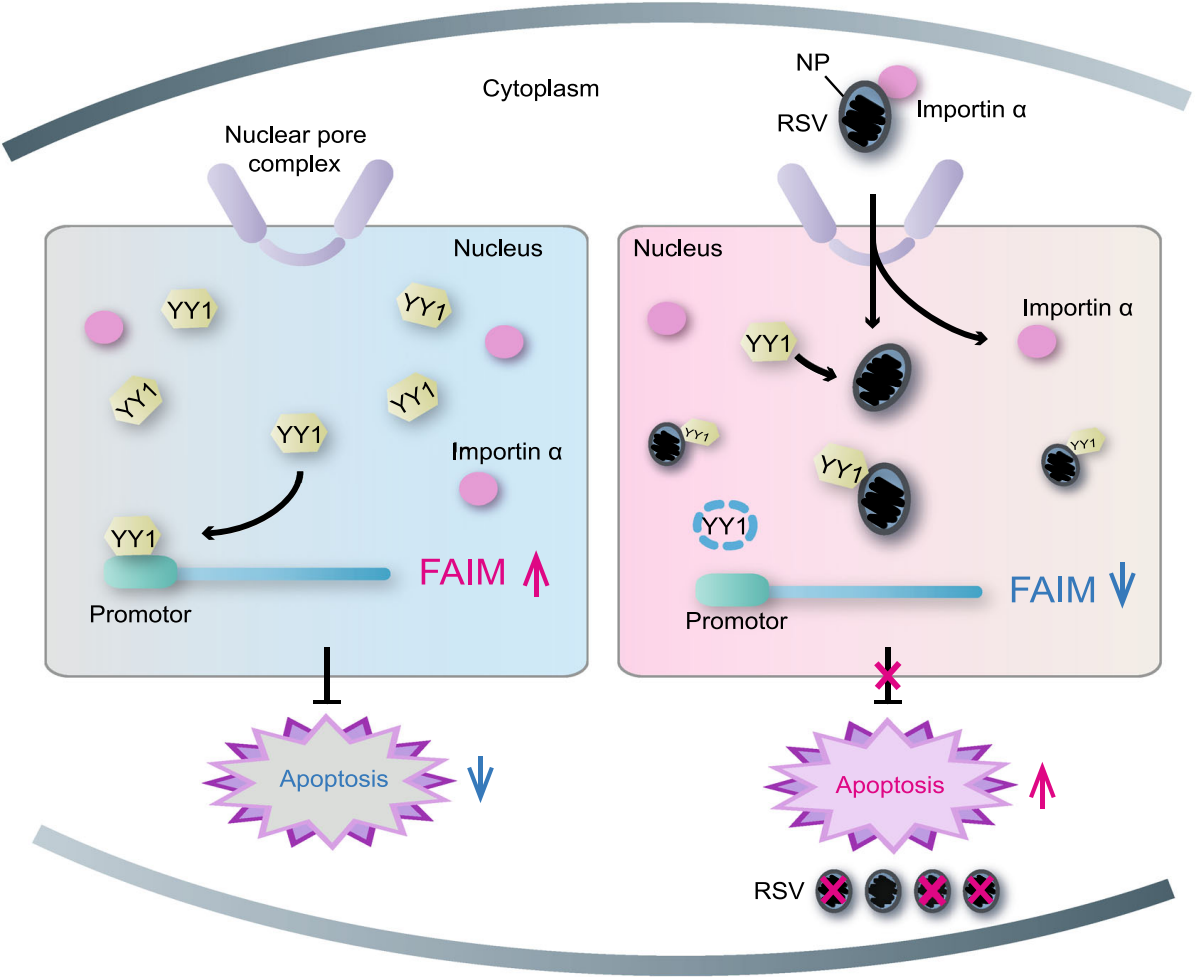
**Model of RSV nuclear entry to inhibit virus replication in vector insect cells**. The nucleocapsid protein (NP) and genomic RNAs of RSV are transported to the nuclei of vector insects through binding to three importin α proteins, which are part of the nuclear transport system of vector cells. In nuclei, NP interacts with the transcription factor YY1 and inhibits the transcriptional regulation of YY1 to *FAIM*. The reduced expression of *FAIM* activates a caspase-dependent apoptotic response, which inhibits viral replication in the cytoplasm

Regarding the RSV and planthopper model system, the results of our study revealed that the structural protein NP and viral genomic RNAs were partially transported into the nuclei of vector insect cells through binding with three importin α proteins, which are the classical nuclear transport system of cells. In the cell nuclei, the interaction between NP and the transcription factor YY1 interfered with the ability of YY1 to positively regulate the expression of *FAIM*, an inhibitor of cell apoptosis. Subsequently, the reduction of *FAIM* expression induced a caspase-dependent apoptotic response, which inhibited viral replication in the cytoplasm (Fig. 7).

This is the first study to report that a plant cytoplasmic RNA virus can enter the nuclei of vector cells, although vector insects belong to evolutionarily divergent hosts for viruses and nuclear localization of RNA viruses in host cells is frequently reported. In our study, the NP and genomic RNAs of RSV were observed in the cell nuclei of vector planthoppers. Some studies previously showed that NP and three non-structural proteins of RSV (NS2, NS3, and SP) were present in the nuclei of host rice and tobacco (Xiong et al., 2009; Kong et al., 2014; Zheng et al., 2018). The genomic RNA of another plant cytoplasmic RNA virus, hibiscus chlorotic ringspot virus, is also located in the nuclei of host cells (Gao et al., 2012). Capsid/nucleocapsid proteins or non-structural proteins entering the host nuclei have been observed in many insect-transmitted RNA viruses, such as the arboviruses dengue virus, Zika virus, and West Nile virus and the plant viruses cucumber mosaic virus and groundnut rosette virus (Ryabov et al., 2004; Wang et al., 2004; Sangiambut et al., 2008; Bhuvanakantham et al., 2009; Grant et al., 2016). Although viral nuclear entry in vector cells has been reported in several arboviruses, such as for the capsid protein C and nonstructural proteins NS1, NS3, and NS5 of dengue serotype 2 virus and the capsid proteins of Mayaro virus and Semliki Forest virus in mosquito cells, their functions were not evaluated (Jakob, 1993; Mitchell et al., 1997; Sangiambut et al., 2008; Hannemann et al., 2013; Reyes-Ruiz et al., 2018). We inferred that this nuclear entry of viral proteins or viruses in vector cells may happen with more insect-transmitted viruses than reported.

Despite the similar manner of viral protein nuclear entry in host and vector cells, the opposite effects are observed on viral replication, reflecting differences in the adaptation strategies of viruses with vectors than with hosts. We observed that the localization of NP activated immune reactions to limit the level of RSV replication in vector cells. The nuclear entry of viruses has been extensively documented to facilitate viral replication, assembly or pathogenicity in host cells. For example, the core protein of hepatitis C virus localizes within cell nuclei to promote persistent infection by specifically modulating host ribosome biogenesis (Bonamassa et al., 2015). In addition, the nonstructural phosphoprotein NS5 of yellow fever virus, Zika virus, and dengue virus localizes in host nuclei and promotes viral replication via suppression of type I interferon transcription (Thurmond et al., 2018). Such different roles of viral nuclear entry in host and vector cells probably related to the different performances of viruses in the two organisms. Viruses must limit replication to maintain a perfect balance between viral load and the immunity pressure in vector cells for efficient transmission, while viruses exhibit excessive replication after countering the immune system in host cells (Blanc and Gutierrez, 2015; Zhao et al., 2016a; Zhao et al., 2018; Zhao et al., 2019). This phenomenon is one of causes for insect-transmitted viruses typically being able to induce serious diseases in hosts but not in vector insects.

We identified a new regulatory mechanism of the YY1 immune pathway. In planthoppers, YY1 positively regulates the expression of *FAIM*, leading to negative regulation of cell apoptosis. In contrast, YY1 takes part in antiviral immunity in host cells by regulating the transcription of *interferon-β* (*IFN-β*) and *IFN-γ*, which stimulate the JAK/STAT signaling pathway (Garcia-Sastre, 2002). The NS protein of Rift Valley fever virus prevents the transcription of *IFN-β* by binding YY1 to facilitate viral replication in host cells (Ly and Ikegami, 2016). Considering that the homologs of mammalian interferons do not exist in insects, we identified a new YY1-regulating downstream gene, *FAIM*, which paves the immune pathway for YY1-mediated cell apoptosis. The activation of cell apoptosis negatively impacts on RSV replication in planthoppers. However, the effects of apoptosis on viral performance in vector insects are controversial. For example, apoptosis is detrimental to Sindbis virus and West Nile virus infection in mosquitoes (Vaidyanathan and Scott, 2006; O’Neill et al., 2015), whereas dengue virus can induce apoptosis to promote viral dissemination in mosquitoes (Eng et al., 2016). Rice ragged stunt virus induces apoptosis in the salivary glands of *N*. *lugens* to promote more efficient transmission (Huang et al., 2015). Rice gall dwarf virus exploits the caspase-dependent apoptotic response in *R*. *dorsalis* to promote viral replication and transmission (Chen et al., 2019). Thus, apoptosis appears to play a complicated role in regulating the relationship between vector and virus.

In summary, for the first time, we observed the nuclear entry phenomenon of a plant cytoplasmic RNA virus in a vector insect. The entry of the viral nucleocapsid protein into the vector cell nuclei results in an antiviral outcome that is quite different from that of viral proteins in host nuclei. The opposite effects of viral nuclear entry reflect different immune adaptation strategies of the insect-transmitted viruses in vector and host cells. Our findings broaden the biological understanding of viral nuclear entry in vector insects.

## Materials and methods

### Small brown planthoppers

Viruliferous and non-viruliferous small brown planthopper strains used in this study were described previously. The viruliferous and non-viruliferous small brown planthopper strains used in this study were obtained from a field population collected at Hai’an, Jiangsu Province, China. The viruliferous strain contained the Jiangsu RSV isolate. Insect rearing was performed as described previously (Zhao et al., 2016a). Another RSV isolate, Yunnan isolate, was collected from Kunming in Yunnan Province and stored at −80°C (Zhao et al., 2018).

### RNA isolation and cDNA synthesis

RNA was isolated from various planthopper samples and nuclear and cytoplasmic protein extracts using TRIzol reagent (Invitrogen, Carlsbad, CA, USA) following the manufacturer’s protocol. After being treated to remove genomic DNA contamination using a TURBO DNA-free kit (Ambion, Austin, TX, USA), 1 μg of RNA was reverse transcribed to cDNA using the Superscript III First-Strand Synthesis System (Invitrogen) and random primers (Promega, Madison, WI, USA) in accordance with the manufacturer’s instructions.

### Sequence alignment and phylogenetic analysis

To identify importin α proteins and caspases in the small brown planthopper, human and *D*. *melanogaster* importin α proteins and caspases as well as *N*. *lugens* caspases were used as queries to search against the planthopper gene set (3) (Zhu et al., 2017) using BLASTp with an E-value cutoff of 1E-5. These sequences were aligned with ClustalW, and neighbor-joining phylogenetic trees were constructed with a bootstrap of 1000 using MegaX. Branches with <50% bootstrap value were collapsed.

### Extraction of nuclear and cytoplasmic proteins and Western blot analysis

Nuclear and cytoplasmic proteins were extracted from viruliferous nymphs using a Nuclear and Cytoplasmic Extraction kit (Beyotime, Jiangsu, China). Thirty nymphs were homogenized in 200 μL of ice-cold cytoplasmic extraction A and B reagents (volume ratio of 20:1) supplemented with a protease inhibitor cocktail (Thermo Fisher Scientific, Waltham, MA, USA) using a TGrinder high-speed tissue grinder (Tiangen Biotech, Beijing, China). After incubating in ice bath for 15 min, the homogenate was centrifuged at 1,500 ×*g* for 5 min at 4 °C. The supernatant was retained as the first cytoplasmic protein fraction. Subsequently, another 200 μL of cytoplasmic extraction reagent A was added to the precipitate. After incubating in ice bath for 15 min, the homogenate was mixed with 10 μL of cytoplasmic extraction reagent B and centrifuged at 12,000 ×*g* for 5 min at 4 °C. The resulting supernatant was retained as the second cytoplasmic protein fraction. Both extracts were then mixed and used as cytoplasmic proteins. Subsequently, the precipitate was mixed with 50 μL of nuclear extraction reagent and vortexed for 30 min. After centrifugation at 12,000 ×*g* for 10 min at 4 °C, the supernatant was retained as the nuclear protein extract fraction. The cytoplasmic and nuclear protein extracts were used for Western blot analysis and RNA extraction. NP was detected using a homemade monoclonal anti-NP antibody. The reference proteins for nuclear and cytoplasmic proteins were histone H3 and GAPDH, which were detected using polyclonal anti-H3 antibody and anti-GAPDH antibody, respectively (Abcam, Cambridge, UK). The optical density of NP was quantified using Gelpro32 image analysis software and was normalized to that of histone H3. Differences were statistically evaluated in SPSS 17.0 using Student’s *t*-test.

### Protein expression, purification and antibody preparation

The open reading frames (ORFs) of importin α1, α2, and α3 and fragments of importin α1 (352 to 528 aa), importin α2 (366 to 524 aa), and importin α3 (350 to 513 aa) were cloned and constructed into the vector pET28a between the restriction sites* Eco*RI and* Xho*I to generate His-tagged recombinant protein expression plasmids. The ORF for YY1 with a His-tag was constructed into pET28a between the restriction sites* Nde*I and* Eco*RI. The RSV ORF for NP with a Flag-tag was inserted into pET28a between the restriction sites *NcoI* and *EcoRI*. Four NP fragments [from 1 to 165 aa (N), 166 to 284 aa (C), 166 to 193 aa (C1), and 204 to 284 aa (C2)] with Flag-tags were inserted into pET28a between the restriction sites* Nde*I and* Eco*RI. The corresponding primers are listed in Table S3. The recombinant plasmids were transformed into Escherichia coli BL21 (DE3) for expression. After 10 h induction with 0.5 mmol/L isopropyl b-D-thiogalactoside (IPTG) at 16 °C, the cells were pelleted by centrifugation and then sonicated for 30 min on ice. Then, the supernatant from the sonicated cells was used for pull down or co-immunoprecipitation assays. The expressed fragments of importin α1, α2 and α3 were purified using Ni Sepharose (GE Healthcare, Buckinghamshire, UK) following the manufacturer’s instructions and served as antigens to produce rabbit anti-importin α1, α2, or α3 polyclonal antibodies by the Beijing Protein Innovation Company (Beijing, China).

### Co-immunoprecipitation for Western blot analysis

For *in vitro* co-immunoprecipitation analysis, approximately 10% of the total recombinant proteins were used as input. To pull down the interacting proteins from the viruliferous planthoppers, total proteins were extracted from 4th-instar viruliferous planthoppers using 1× PBS buffer (pH 7.2) supplemented with a protease inhibitor cocktail (Thermo Fisher Scientific). Approximately 10% of the total protein fraction was reserved as input. Then, 10 μL of protein G beads (Thermo Fisher Scientific) was mixed with 2 μg of an anti-His/-Flag monoclonal antibody (Merck Millpore, Billerica, MA, USA) or anti-YY1 polyclonal antibody (Thermo Fisher Scientific) or a homemade anti-NP monoclonal antibody before being incubated with 200 μL of His- or Flag-tagged recombinant proteins for 15 min at room temperature. The expression products from the pET28a vector or the IgG antibody (Merck Millipore) were used as negative controls. Then, 500 μL of recombinant Flag- or His-tagged target proteins or the total proteins from viruliferous planthoppers were added and incubated at 4 °C overnight. Finally, the antibody-protein-protein complex was dissociated from the beads with the elution buffer (Thermo Fisher Scientific) for Western blot analysis.

### Co-immunoprecipitation and mass spectrometry for identification of NP-interacting nuclear proteins

Protein G beads (10 μL) were mixed with 2 μg of a monoclonal anti-NP or IgG antibody (Merck Millipore) and then incubated with 500 μL of nuclear protein extracts from viruliferous planthoppers. Then, the antibody-protein-protein complex was dissociated from the beads with elution buffer (Thermo Fisher Scientific) for liquid chromatography-tandem mass spectrometry with a Q-Exactive instrument (Thermo Fisher Scientific) by the Beijing Protein Innovation Company. The mass spectrometry data were searched against the genome database of the small brown planthopper (Zhu et al., 2017). Peptides with scores higher than 80 were screened as putative NP-interacting nuclear proteins for further analysis.

### Protein expression in S2 cells

The NP ORF was cloned into the plasmid pAc-5.1/V5-HisB (Invitrogen). The C2 fragment of NP (204 to 284 aa) and GFP were cloned into the plasmid pIEx-4 between the restriction sites* Kpn*I and* Not*I to yield the plasmid pIEx-4-GFP-Gly-Ser (linker)-C2. The primers used for cloning are listed in Table S3, and the plasmids pAc-5.1/V5-HisB and pIEx-4-GFP were used as controls. For each plasmid, 20 ng was transformed into Drosophila S2 cells using Lipofectamine 3000 reagent (Thermo Fisher Scientific). The cells were sampled at 45 h after transfection and fixed with 4% (*w*/*v*) paraformaldehyde for 30 min. For NP-expressing cells, the monoclonal anti-NP antibody and Alexa Fluor 488 (green) affinipure goat anti-mouse IgG (YEASEN, Shanghai, China) were sequentially added. For GFP- and GFP-C2-expressing cells, GFP signals were directly observed. Nuclei were labeled with Hoechst (blue), and fluorescence was examined under a Leica TCS SP5 confocal microscope (Leica Microsystems, Solms, Germany).

### Immunohistochemistry analysis

Midguts or salivary glands from viruliferous, non-viruliferous or dsRNA-treated planthoppers were dissected in 1× PBS buffer (pH 7.2) on a glass plate and fixed in 4% (*w*/*v*) paraformaldehyde for 2 h at room temperature. After being treated with osmotic buffer (0.01 mol/L phosphate-buffered saline containing 2% Triton X-100, pH 7.4) for 2 h, the tissues were incubated with the monoclonal anti-NP antibody and/or the polyclonal anti-importin α3 antibody overnight at 4 °C. After being washed with 0.01 mol/L phosphate-buffered saline containing 2% Tween-20 (pH 7.4), the tissues were blocked with 3% bovine serum albumin for 1 h. Then, the secondary antibody Alexa Fluor 488 (green) affinipure goat anti-mouse IgG or Alexa Fluor 594 (red) affinipure goat anti-rabbit IgG (YEASEN) was added. The nuclei were labeled with Hoechst (blue). The samples without the treatment of primary antibodies were used as negative controls. The images were viewed under a Leica TCS SP5 confocal microscope (Leica Microsystems, Solms, Germany).

### Fluorescence *in situ* hybridization

The localization of RSV genomic RNAs in planthopper salivary glands and guts were performed using an RNA3 probe labeled with digoxigenin (DIG). The probe for RNA3 was synthetized using a T7/SP6 RNA Transcription kit (Roche, Basel, Switzerland) and was subsequently fragmented to approximately 250 bp via the alkaline lysis method. The primers used for RNA3 probe synthesis are listed in Table S3. Salivary glands and guts were dissected from viruliferous planthoppers and then fixed in 4% (*w*/*v*) paraformaldehyde at 4 °C overnight. After being digested with 20 μg/mL of proteinase K at 37 °C for 15 min, tissues were hybridized with 5 ng/μL of RNA3 probe at 37 °C overnight and then successively washed in 2×, 1×, and 0.2× SSC at 37 °C for 30 min twice. An anti-DIG alkaline phosphatase-conjugated antibody (1:500) was used for RNA3 probe detection. The DIG fluorescent signal was detected under an HNPP Fluorescent Detection Set (Roche). The samples without the treatment of RNA3 probe were used as negative controls. Images were viewed under a Leica TCS SP5 confocal microscope (Leica).

### Colloidal gold immunoelectron microscopy

The midguts of viruliferous and nonviruliferous planthoppers were fixed with 4% paraformaldehyde and 0.5% glutaraldehyde in 0.1 mol/L phosphate buffer (pH 7.4) at 4 °C overnight. After dehydration in 30%, 50%, 70%, 85%, 95% and 100% alcohol, the midguts were embedded in LR Gold Resin (Fluka Biochemika, Steinheim, Switzerland). Then, 70 nm-ultrathin sections of the embedded tissues were cut and placed onto 50-mesh copper grids before being blocked for 1 h in 100 mmol/L PBS containing 5% goat serum. The anti-NP monoclonal antibody (1:200) and 10 nm gold-conjugated goat-anti-mouse IgG (1:100) were sequentially added for 1.5 h incubations. Another group of sections from viruliferous insects was not treated with the anti-NP monoclonal antibody as a negative control. After being washed with PBS buffer five times, the grids were stained with 2% neutral uranyl acetate for 10 min in the dark. Then, the sections were viewed under a JEM-1400 transmission electron microscope (JEOL, Tokyo, Japan) at an accelerating voltage of 80 kV. Six sections of fifteen midguts from viruliferous or nonviruliferous insects were observed.

### Double-stranded RNA synthesis and delivery

Double-stranded RNAs (dsRNAs) for *importin α-1*, *2* and *3*, *YY1*, *FAIM*, and *green fluorescent protein* (*GFP*) were synthesized using the T7 RiboMAX Express RNAi System (Promega) following the manufacturer’s protocol. The corresponding PCR primers of dsRNA for these genes are shown in Table S3. A total volume of 23 nL of dsRNA at 6 μg/μL for each gene were delivered into the third-instar nymphs through microinjection using a Nanoliter 2000 instrument (World Precision Instruments, Sarasota, FL, USA). The corresponding primers of dsRNA for these genes are shown in Table S3.

### Inoculation of small brown planthoppers with RSV crude preparations

RSV crude preparations were extracted from 50 viruliferous adult planthoppers with 100 μL of PBS buffer (pH 7.2) as previously described (Zhao et al., 2016a). Non-viruliferous fourth-instar nymphs were injected with 23 nL of a mixture of insect-derived RSV crude preparations and 6 μg/μL ds*YY1*-RNA using a Nanoliter 2000 instrument (World Precision Instruments). The control group was injected with a mixture of RSV crude extracts and ds*GFP*-RNA. After the injection, the planthoppers were raised on healthy rice seedlings.

### Quantitative real-time PCR and reverse transcription-PCR

Quantitative real-time PCR (qPCR) was used to measure the relative RNA levels of *NP* and the genomic RNA3 of RSV and planthopper genes on a Light Cycler 480 II instrument (Roche). The primers for each gene are listed in Table S3. qPCR was performed in a volume of 10 μL comprising 1 μL of template cDNA, 5 μL of 2× SYBR Green PCR Master Mix (Fermentas, Waltham, MA, USA), and 0.25 μL of each primer (10 μmol/L). The thermal cycling conditions were 95 °C for 2 min followed by 45 cycles of 94 °C for 20 s, 60 °C for 30 s and 72 °C for 10 s. The PCR products were from 80 to 150 bp. Five to eight insects or 15 to 18 tissues per replicate and six to eight biological replicates were prepared. The transcript level of planthopper translation elongation factor 2 (EF2) was quantified to normalize the cDNA templates. The relative transcript level of each gene to that of *EF2* was reported as the mean ± SE. Differences were statistically evaluated using Student’s *t*-test to compare the two means and one-way ANOVA followed by a Tukey’s test for multiple comparisons.

The four genomic RNAs of RSV were examined in the nuclear and cytoplasmic protein extracts by reverse transcription-PCR (RT-PCR) as described previously (Zhao et al., 2018) using the primers listed in Table S3. The PCR protocol was 94 °C for 3 min followed by 30 cycles of 94 °C for 30 s, 55 °C for 30 s, and 72 °C for 30 s, with a final incubation at 72 °C for 5 min.

### Target prediction of YY1 and NLS prediction of NP

The binding sequences of the transcription factor YY1 were predicted in the promoter regions of the small brown planthopper genes (Zhu et al., 2017). Each promoter sequence was regarded as the 2,000-bp sequence upstream of the transcription start site of each gene. We predicted the potential targets of YY1 using the algorithms PROMO with the TRANSFAC 8.3 database (http://alggen.lsi.upc.es) based on the conservative YY1 binding motif (5′-CCGCCATNTT-3′) (Kim and Kim, 2009). Six candidate targets relative to the immune responses were verified via qPCR and ChIP-qPCR. The NLS of NP was predicted using cNLS mapper (http://nls-mapper.iab.keio.ac.jp/cgi-bin/NLS_Mapper_form.cgi) with a cutoff score of 2.

### ChIP-qPCR analysis

ChIP was performed using a SimpleChIP plus Enzymatic Chromatin IP kit (Cell Signaling Technology, Danvers, MA, USA) in accordance with the manufacturer’s instructions. One hundred fifth-instar nymphs from nonviruliferous planthoppers were crushed and treated with 1.5% formaldehyde for 15 min before being incubated with 125 mmol/L glycine for 5 min. Then, 1× buffer A containing 0.5 mmol/L DTT and 1× protease inhibitor cocktail (PIC) were added. The DNA was digested with 100 µL of micrococcal nuclease (1× buffer B, 0.5 mmol/L DTT, 1× PIC, and 0.5 µL of micrococcal nuclease) for 20 min at 37 °C and then incubated with 5 mmol/L EDTA for 10 min on ice. The pelleted nuclei were sonicated with three cycles of 20 s on and 30 s off. After centrifugation, the supernatant was diluted with 1× ChIP buffer (containing 1× PIC), and 10 µL of the diluted samples was set aside as the 2% input DNA. Immunoprecipitation was performed using anti-histone H3 (D2B12; Cell Signaling Technology), normal rabbit IgG (Cell Signaling Technology), or anti-YY1 polyclonal antibody (Thermo Fisher Scientific) together with ChIP-Grade Protein G Magnetic Beads at 4 °C overnight. After sequentially being washed with low salt buffer three times, high salt buffer three times, and TE buffer two times, DNA was eluted in 1× ChIP elution buffer for 30 min at 65 °C followed by addition of proteinase K and another 2 h incubation at 65 °C. The ChIP-enriched DNA was purified using spin columns following the manufacturer’s instructions before being used for qPCR analysis on a Light Cycler 480 II instrument (Roche). The promoter regions of six putative target genes of YY1 were amplified via qPCR using specific primers (Table S3). The promoter region of GAPDH1 was quantified in the anti-histone H3 immunoprecipitation as positive control. The thermal cycling conditions were as follows: 95 °C for 3 min followed by 40 cycles of 95 °C for 15 s and 60 °C for 60 s. The qPCR results were analyzed using the percent input method, the equation for which is shown below. Using this method, signals obtained from each immunoprecipitation are expressed as a percent of the total input chromatin. ChIP-qPCR was repeated three times.

$${\text{Percent}}\;{\text{Input }} = \, 2\% \, \times \, 2^{{({\text{CT }}\,2\% \;{\text{Input}}\;{\text{Sample }}{-}{\text{ CT}}\;{\text{IP}}\;{\text{Sample}})}}$$

$${\text{CT }} = {\text{ Threshold}}\;{\text{cycle}}\;{\text{of}}\;{\text{PCR}}$$

To compare the amount of *FAIM* promoter DNA that bound with YY1 from viruliferous and nonviruliferous planthoppers, ChIP was performed in viruliferous and nonviruliferous nymphs as described above. After quantifying the YY1 protein level using ELISA (Zhao et al., 2016a) and Western blot assay, the DNA contained in the comparable amounts of YY1 was extracted and the FAIM promoter was quantified with qPCR using specific primers (Table S3). IgG was used instead of the anti-YY1 antibody in the immunoprecipitation step as the negative control. Eight biological replicates were prepared for each group. The relative enrichment of *FAIM* promoter DNA binding with YY1 to that of IgG immunoprecipitation group (fold enrichment) were compared between viruliferous and nonviruliferous planthoppers and the difference was statistically evaluated using Student’s *t*-test.

### Determination of caspase activity in planthoppers

Caspase activities were determined in viruliferous or nonviruliferous four-instar nymphs using human caspase 1, 3 and 8 Activity Assay kits (Beyotime Biotechnology) and a SpectraMax® Paradigm® Multi-Mode Detection Platform (Molecular Devices, San Jose, CA, USA) according to the manufacturer’s instructions.

Standard curves were constructed based on the molarities of p-nitroaniline (pNA) and OD405 values. The specific substrate was Ac-YVAD-pNA for caspase 1, Ac-DEVD-pNA for caspase 3, and Ac-IETD-pNA for caspase 8. Caspase activities were calculated as the product pNA (µmol/L). The slope and square of the correlation coefficient (R2) of each standard curve was calculated. The activities were measured at 7 d after the injection of dsRNAs. Twenty nymphs were used in one replicate, and four to six replicates were prepared for each group.

### Terminal deoxynucleotidyl transferase dUTP nick-end labeling (TUNEL) assay

Midguts were dissected from three groups of planthoppers: viruliferous and nonviruliferous four-instar nymphs; nymphs 7 d after dsRNA-injection; and nymphs 6 d after the injection of 13.8 nL of 25 μmol/L pan-caspase inhibitor Z-VAD-FMK (Promega) or dimethyl sulfoxide (DMSO), performed in duplicate. After being fixed in 4% (*w*/*v*) paraformaldehyde and treated with the osmotic buffer, the midguts were used for TUNEL staining with a One Step TUNEL Apoptosis Assay kit (Beyotime Biotechnology) following manufacturer’s instructions. NP was assessed using the monoclonal anti-NP antibody in immunohistochemistry assays as described above. Fluorescence images were examined using a Leica TCS SP5 confocal microscope (Leica). The midguts treated with the pan-caspase inhibitor or DMSO were also sampled to quantify the RNA levels of NP and the genomic RNA3 of RSV via qPCR. Eight biological replicates and 15 to 18 midguts per replicate were prepared for qPCR.

## Supplementary Information

Below is the link to the electronic supplementary material.

Supplementary material 1 (PDF 1094 kb)

## Data Availability

All data generated or analysed during this study are included in this published article (and its supplementary information files).

## Abbreviations

ChIP-qPCR: chromatin immunoprecipitation coupled with qPCR
Co-IP: co-immunoprecipitation
Cy: cytoplasmic
DIG: digoxigenin
dsRNAs: double-stranded RNAs
FAIM: Fas apoptotic inhibitory molecule
FISH: fluorescent in situ hybridization
GFP: green fluorescent protein
IMPα: importin α
Nc: nucleus
NLS: nuclear localization signals
NP: nucleocapsid protein
qPCR: quantitative real-time PCR
RIP: RNA immunoprecipitation
RSV: rice stripe virus
RT-PCR: reverse transcription-PCR
TUNEL: terminal deoxynucleotidyl transferase dUTP nick-end labeling assay
YY1: transcription factor Yin Yang-1
