# Correction to: The nucleocapsid protein of rice stripe virus in cell nuclei of vector insect regulates viral replication

**DOI:** 10.1007/s13238-021-00854-7

**Published:** 2021-09-01

**Authors:** Wan Zhao, Junjie Zhu, Hong Lu, Jiaming Zhu, Fei Jiang, Wei Wang, Lan Luo, Le Kang, Feng Cui

**Affiliations:** 1 State Key Laboratory of Integrated Management of Pest Insects and Rodents, Institute of Zoology, Chinese Academy of Sciences, Beijing, 100101 China; 2 CAS Center for Excellence in Biotic Interactions, University of Chinese Academy of Sciences, Beijing, 100049 China

## Correction to: Protein Cell 10.1007/s13238-021-00822-1

In the original publication of the article the fill color and the border color of the histograms in Fig. 3A (dsGFP and dsIMPa1) and Fig. 3B were incorrect. Also, the schematic plot of the N fragment of NP in Fig. 2D was not clear enough. The correct Figs. 2 and 3 are provided in this correction.

**Figure 2:**
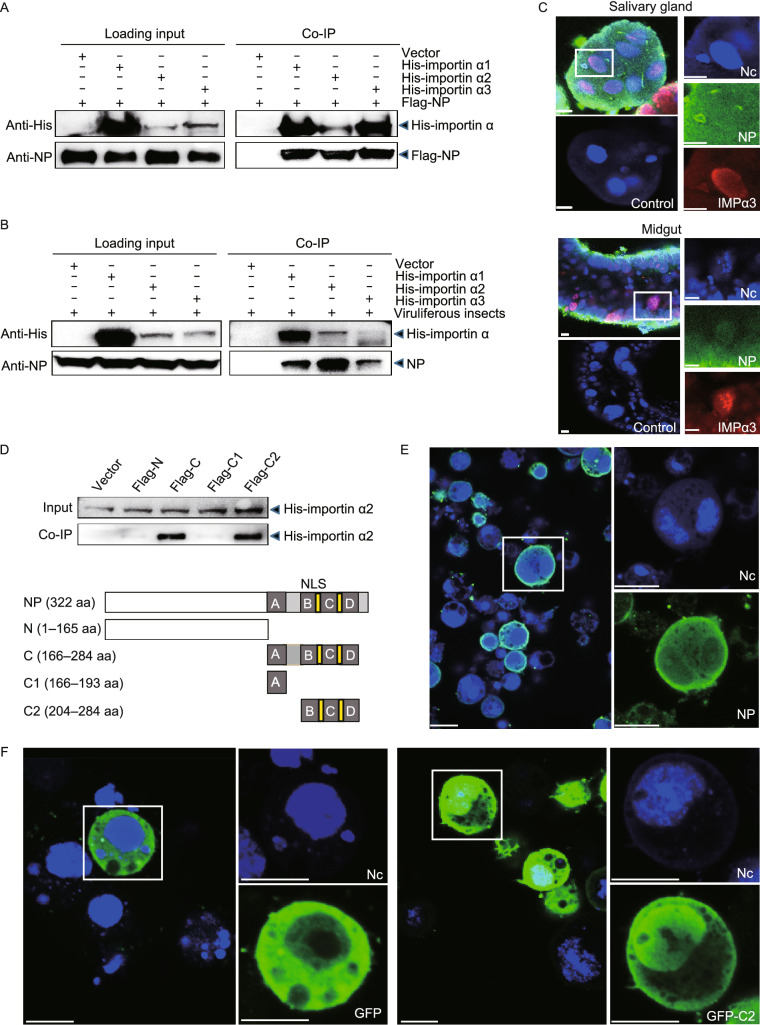
**RSV nucleocapsid protein interacts with importin α proteins via.** **the nuclear localization signal.** (A) Recombinantly expressed RSV nucleocapsid protein (NP) with a Flag-tag binds three recombinantly expressed His-importin α proteins in the co-immunoprecipitation (Co-IP) and Western blot assay. The expression products from the pET28a vector were used as a negative control. (B) Three recombinantly expressed His-importin α proteins pulled down the NP from viruliferous planthoppers in the Co-IP and Western blot assay. The expression products from the pET28a vector were used as a negative control. (C) Colocalization of importin α3 and NP examined in the salivary gland and midgut cells via immunohistochemistry analysis. The green signal is from an Alexa Fluor 488-labeled anti-NP monoclonal antibody. The red signal is from an Alexa Fluor 594-labeled anti-importin α3 (IMPα3) polyclonal antibody. The boxed region is enlarged and shown in three different panels on the right side. The samples without the treatment of primary antibodies are shown as negative controls. (D) Co-IP and Western blot assay for the interaction between the recombinantly expressed Flag-N, Flag-C, Flag-C1, or Flag-C2 fragments of NP and His-importin α2. Motifs A, B, C and D comprise the putative nuclear localization signals (NLS). The overlapping amino acid residues shared by the motifs are marked in yellow. The expression products from the pET28a vector were used as a negative control. (E) Immunohistochemistry analysis of the subcellular location of expressed NP in S2 cells. The green signal is from an Alexa Fluor 488-labeled anti-NP monoclonal antibody. The boxed region is enlarged and shown in two different panels on the right side. (F) Subcellular location of expressed GFP and the GFP-C2 fragment in S2 cells was observed. The boxed region is enlarged and shown in two different panels on the right side. Scale bars in 2C, 2E, and 2F: 10 μm. The blue signal is the nuclei (Nc) stained with Hoechst

**Figure 3:**
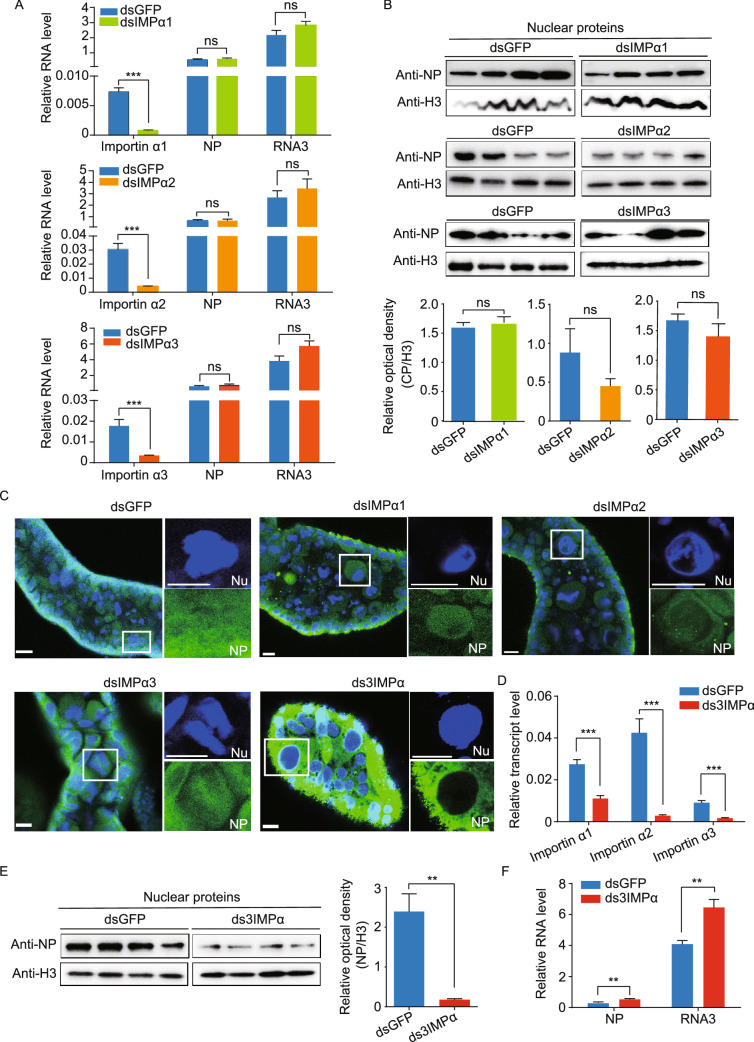
**Inhibition of NP nuclear entry significantly promotes RSV accumulation in planthoppers**. (A) Relative RNA levels of RSV *NP* and genomic RNA3 7 d after the injection of dsRNA of *importin α1* (dsIMPα1), *importin α2* (dsIMPα2), *importin α3* (dsIMPα3), or *GFP* (dsGFP), as measured by quantitative real-time PCR (qPCR). The RNA levels of *NP* and RNA3, and the transcript levels of *importin α* genes are normalized to that of *EF2*. (B) The nuclear accumulation of NP in the nuclear protein extracts of viruliferous planthoppers after the injection of dsIMPα1, dsIMPα2, dsIMPα3 or dsGFP for 7 d, as assessed by Western blot assay. NP was detected using a monoclonal anti-NP antibody. The reference protein for the nuclear proteins was histone H3, which was detected using a monoclonal anti-H3 antibody. Four independent replicates are shown for each group. The relative optical densities of NP to that of H3 were calculated. (C) Immunohistochemistry analysis of the subcellular location of NP in the midgut cells after the injection of dsIMPα1, dsIMPα2, dsIMPα3, a mixture of dsRNA of the three *importin α* genes (ds3IMPα), or dsGFP for 7 d. NP was detected using a monoclonal anti-NP antibody. The blue signal is the nuclei (Nc) stained with Hoechst. The boxed region is enlarged and shown in two different panels on the right side. Scale bars: 20 μm. (D) The relative transcript levels of *importin α1*, *α2* and *α3* after the injection of ds3IMPα or dsGFP for 7 d measured by qPCR. (E) Nuclear accumulation of NP in the nuclear protein extracts of viruliferous planthoppers after the injection of ds3IMPα or dsGFP for 7 d, as assessed via Western blot assay. Four independent replicates are shown for each group. The relative optical densities of NP to that of H3 are calculated. (F) Relative RNA levels of RSV *NP* and genomic RNA3 after injection of ds3IMPα or dsGFP for 7 d measured by qPCR. ns, no significant difference. **P < 0.01. ***P < 0.001

