# Supplementary material for: The nucleocapsid protein of rice stripe virus in cell nuclei of vector insect regulates viral replication
Source: Protein Cell. 2021 Mar 6;13(5):360–78. doi: 10.1007/s13238-021-00822-1 (PMC7936609; doi:10.1007/s13238-021-00822-1)
Supplement: Supplementary file 1 — Supplementary material 1 (PDF 1094 kb) [file 13238_2021_822_MOESM1_ESM.pdf]

Supplementary materials for

The nucleocapsid protein of rice stripe virus in cell nuclei of vector insect regulates viral replication

Wan Zhao[#1,2], Junjie Zhu[#1,2], Hong Lu[1,2], Jiaming Zhu[1,2], Fei Jiang[1,2], Wei Wang[1,2], Lan Luo[1,2], Le Kang[1,2], Feng Cui[*1,2]

Feng Cui
Tel: +86-10-64807218, Email: cuif@ioz.ac.cn.

This file includes:

Figures S1 to S5
Tables S1 to S3

Figure S1

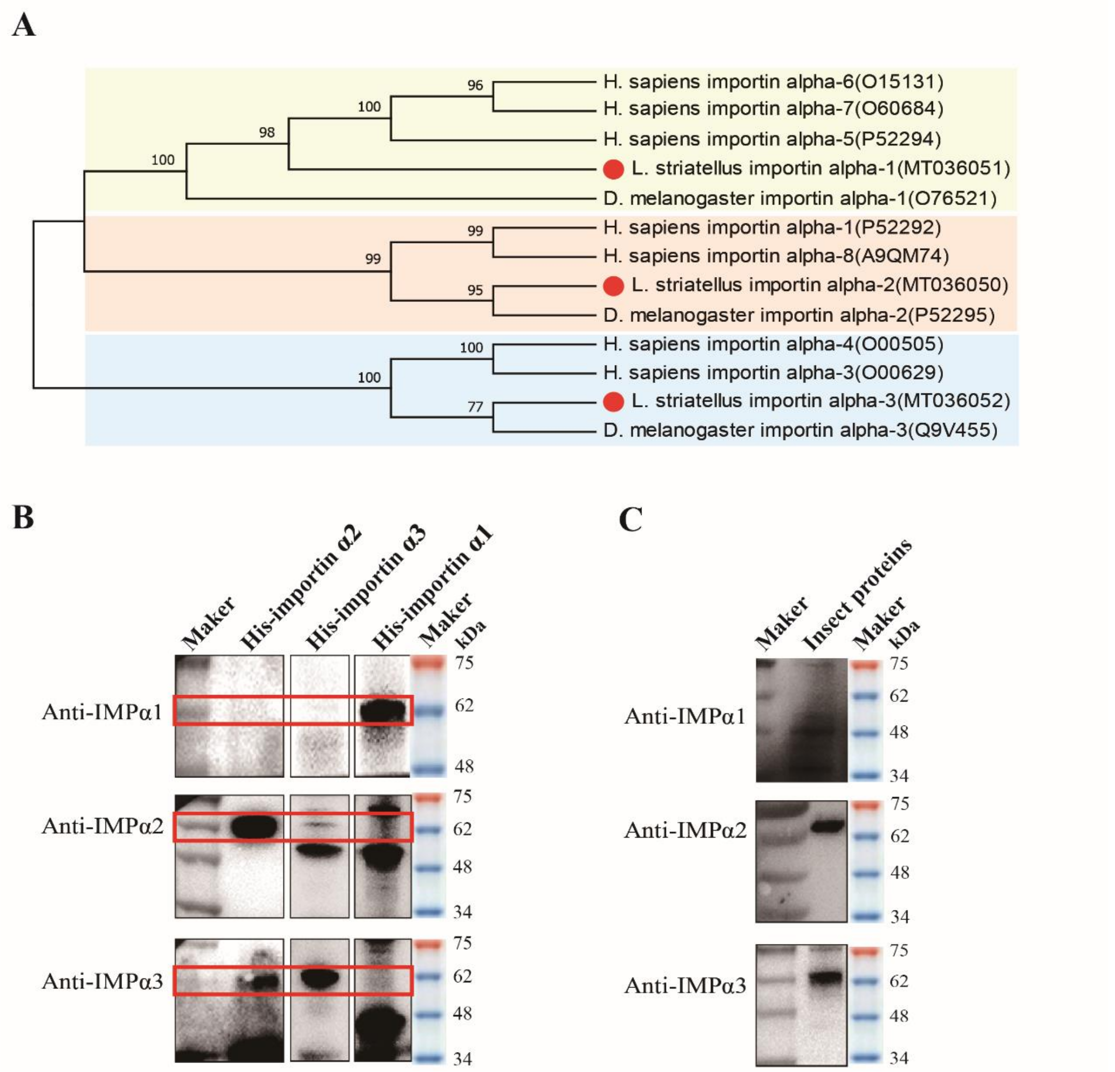

Figure S1. Analysis of importin α1, α2 and α3 from the small brown planthopper. (A) Neighbor-joining phylogenetic tree showing the evolutionary relationships of the three importin α proteins of the small brown planthopper with the importin α proteins of human and *Drosophila melanogaster*. Branches with < 50% bootstrap value are collapsed. (B) Verification of anti-importin α1 (anti-IMPα1), α2 (anti-IMPα2), and α3 (anti-IMPα3) polyclonal antibodies using in vitro expressed recombinant His-importin α proteins via western blot. The red boxes indicate the positions of target proteins. (C) Verification of anti-IMPα1, anti-IMPα2, and anti-IMPα3 polyclonal antibodies using the total proteins of viruliferous planthoppers by western blot.

Figure S2

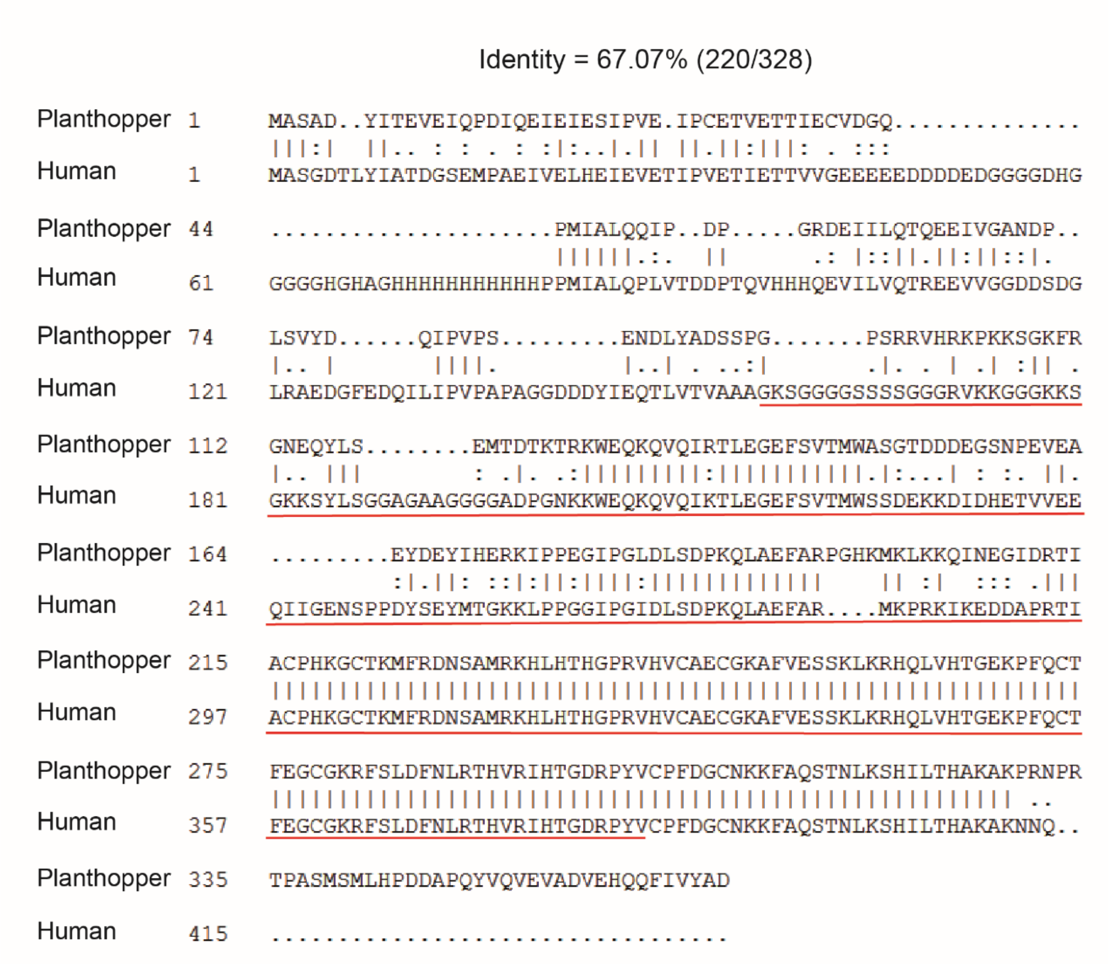

Figure S2. Amino acid identity of planthopper YY1 to human YY1. Identical amino acids are indicated by short vertical lines. The antigen region producing the anti-human YY1 polyclonal antibody is underlined in red.

Figure S3

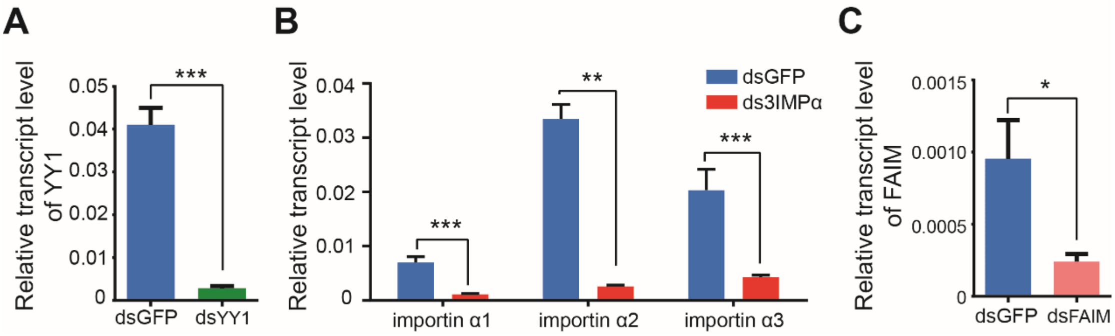

Figure S3. Knockdown efficiencies of dsRNA injection measured by quantitative real-time PCR. (A) Relative transcript levels of *YY1* to that of *EF2* in nonviruliferous planthoppers at 7 d after injection of dsRNA of *YY1* (ds*YY1*) or *GFP* (ds*GFP*). (B) Relative transcript levels of the three *importin α* genes to that of *EF2* in viruliferous insects at 7 d after injection of dsRNA mixture of the three *importin α* genes (ds*3IMPα*) or ds*GFP*. (C) Relative transcript levels of *FAIM* to that of *EF2* in nonviruliferous insects at 7 d after injection of dsRNA of *FAIM* (ds*FAIM*) or ds*GFP*. *, $P < 0.05$. **, $P < 0.01$. ***, $P < 0.001$.

Figure S4

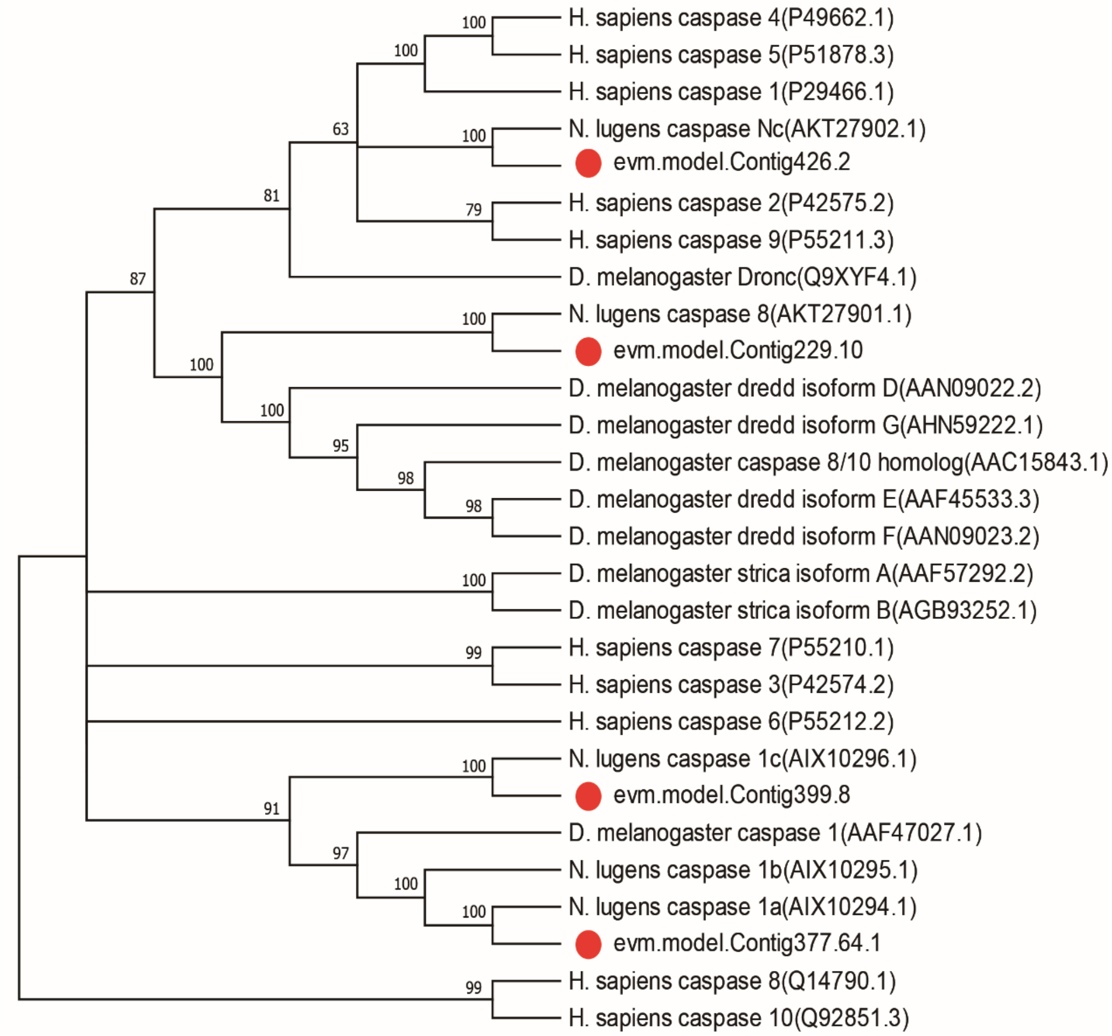

Figure S4. Neighbor-joining phylogenetic tree showing the evolutionary relationships of the small brown planthopper caspases with the caspases of human, *Drosophila melanogaster*, and *Nilaparvata lugens*. The red dots indicate the small brown planthopper caspases. Branches with < 50% bootstrap value are collapsed.

Figure S5

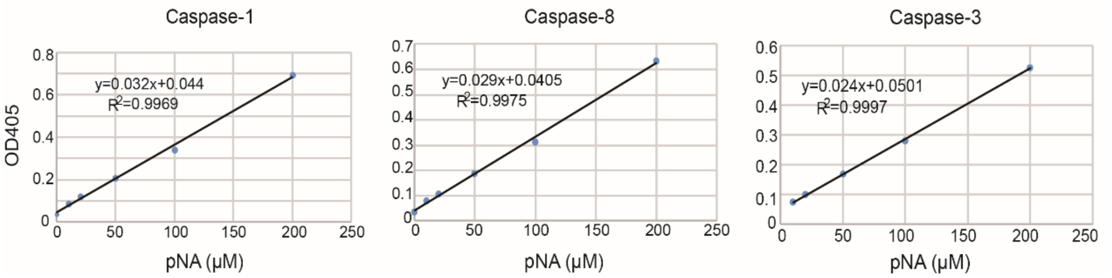

Figure S5. Standard curves for caspase activity measurement. Standard curves were constructed based on the molarities of the product p-nitroaniline (pNA) and OD_{405} values using human caspase 1, 3 and 8 Activity Assay kits. The specific substrate was Ac-YVAD-pNA for caspase 1, Ac-DEVD-pNA for caspase 3, and Ac-IETD-pNA for caspase 8. The slope and square of the correlation coefficient (R^2) of each standard curve was calculated.

Table S1. NP-interacting nuclear proteins identified by Co-immunoprecipitation and mass spectrometry assays.

Accession number in the genome of the small brown planthopper	Annotation
**Ribosomal proteins (30)**	
evm.model.Contig100.148	40S ribosomal protein S26 [Zootermopsis nevadensis]
evm.model.Contig1005.21	ribosomal protein S23e [Graphocephala atropunctata]
evm.model.Contig118.11	60S ribosomal protein L15 [Tribolium castaneum]
evm.model.Contig13.58	60S ribosomal protein L44 [Tribolium castaneum]
evm.model.Contig136.13	acidic ribosomal protein [Ceratitis capitata]
evm.model.Contig139.15	60S ribosomal protein L23 [Apis mellifera]
evm.model.Contig140.5	40S ribosomal protein S16 [Apis mellifera]
evm.model.Contig192.86	ribosomal protein L6 [Riptortus pedestris]
evm.model.Contig2.6	ribosomal protein 49 [Graphocephala atropunctata]
evm.model.Contig219.91	40S ribosomal protein S13 [Coptotermes formosanus]
evm.model.Contig223.12	ribosomal protein L27e [Dascillus cervinus]
evm.model.Contig235.20	ribosomal protein L35 [Riptortus pedestris]
evm.model.Contig25.64	ribosomal protein L28 [Riptortus pedestris]
evm.model.Contig3.18	60S ribosomal protein L21 [Apis mellifera]
evm.model.Contig30.233	ribosomal protein L18A [Graphocephala atropunctata]
evm.model.Contig353.20	ribosomal protein L7 [Riptortus pedestris]
evm.model.Contig430.8	60S ribosomal protein L17 [Zootermopsis nevadensis]
evm.model.Contig445.4	60S ribosomal protein L7a [Laodelphax striatella]
evm.model.Contig47.22	ribosomal protein S4e [Graphocephala atropunctata]
evm.model.Contig615.4	40S ribosomal protein S15a [Zootermopsis nevadensis]
evm.model.Contig64.112	S18e ribosomal protein [Cicindela campestris]
evm.model.Contig649.9	60S ribosomal protein L5 [Laodelphax striatella]
evm.model.Contig679.11.1	60S ribosomal protein L8 [Laodelphax striatella]
evm.model.Contig8.267	40S ribosomal protein S14 [Zootermopsis nevadensis]
evm.model.Contig8.325	60S ribosomal protein L12 [Pediculus humanus corporis]
evm.model.Contig84.1	ribosomal protein L19e [Coptotermes formosanus]
evm.model.Contig84.3	ribosomal protein L11 [Triatoma infestans]
evm.model.Contig905.5	60S ribosomal protein L30 [Zootermopsis nevadensis]
evm.model.Contig94.3	ribosomal protein S17e [Diaphorina citri]
evm.model.Contig970.1	60S ribosomal protein L9 [Sogatella furcifera]
**Heat shock proteins (4)**	
evm.model.Contig118.15	heat shock cognate protein 70 [Nilaparvata lugens]
evm.model.Contig25.171	heat shock protein 90 [Laodelphax striatella]
evm.model.Contig79.49	10 kDa heat shock protein [Lygus hesperus]
evm.model.Contig98.76	heat shock cognate protein 70 [Laodelphax striatella]
**ATP related proteins (12)**	

evm.model.Contig101.39	ATP synthase subunit gamma, mitochondrial [Zootermopsis nevadensis]
evm.model.Contig109.69	ATP-dependent RNA helicase me31b [Athalia rosae]
evm.model.Contig152.40	ATPase [Homo sapiens]
evm.model.Contig18.7	ATP citrate lyase isoform X1 [Acyrthosiphon pisum]
evm.model.Contig385.20.6	sarco/endoplasmic reticulum calcium ATPase [Bombyx mori]
evm.model.Contig39.91	Na+, K+ ATPase alpha-subunit 1 [Boisea trivittata]
evm.model.Contig427.12	ADP/ATP translocase [Nilaparvata lugens]
evm.model.Contig449.18	ATP-dependent RNA helicase p62 [Tribolium castaneum]
evm.model.Contig54.43	transitional endoplasmic reticulum ATPase TER94 isoform X1 [Cerapachys biroi]
evm.model.Contig596.9	obg-like ATPase 1 [Apis florea]
evm.model.Contig65.109	ATP synthase oligomycin sensitivity conferral protein [Graphocephala atropunctata]
evm.model.Contig101.39	ATP synthase subunit gamma, mitochondrial [Zootermopsis nevadensis]
**Cytoskeletion (9)**	
evm.model.Contig136.11	tubulin alpha-2 [Laodelphax striatella]
evm.model.Contig92.25	myosin RLC2 [Nilaparvata lugens]
evm.model.Contig500.4	myosin light chain kinase, smooth muscle-like [Diaphorina citri]
evm.model.Contig640.9.1	titin isoform X3 [Bombus terrestris]
evm.model.Contig108.8	alpha-actinin, sarcomeric isoform X1 [Acyrthosiphon pisum]
evm.model.Contig355.2	hypothetical protein FF38_04907 [Lucilia cuprina]
evm.model.Contig32.134	troponin I [Nilaparvata lugens]
evm.model.Contig108.14.1	myofilin isoform a [Acyrthosiphon pisum]
evm.model.Contig136.11	tubulin alpha-2 [Laodelphax striatella]
Transcriptional factors (1)	
evm.model.Contig26.112	transcriptional repressor protein YY1 [Zootermopsis nevadensis]
**Histone (1)**	
evm.model.Contig109.42	histone H4 [Saimiri boliviensis boliviensis]
Oxidases (4)	
evm.model.Contig109.22	cytochrome c oxidase,-subunit VIb [Riptortus pedestris]
evm.model.Contig112.13	prophenoloxidase [Riptortus pedestris]
evm.model.Contig642.5	NADH-ubiquinone oxidoreductase 39 kda subunit [Anopheles darlingi]
evm.model.Contig84.87	multicopper oxidase 3 [Nilaparvata lugens]

Table S2. Putative target genes of YY1 containing the YY1 binding motif in the promoter region.

Accession number in the genome of the small brown planthopper	Subject Start	Subject End	Bit Score	E-value	Annotation
evm.model.Contig1129.9#Contig1129#176539#178539	1	499	809	0	brahma-associated protein of 60 kDa isoform X1 [Vollenhovia emeryi]
evm.model.Contig692.10#Contig692#286878#288878	20	556	933	0	T-complex protein subunit alpha [Locusta migratoria]
evm.model.Contig256.14#Contig256#451132#453132	74	917	950	0	epidermal cell surface receptor [Zootermopsis nevadensis]
evm.model.Contig170.38#Contig170#797422#799422	1	385	541	0	cAMP-dependent protein kinase type II regulatory subunit [Zootermopsis nevadensis]
evm.model.Contig1635.2#Contig1635#32713#34713	2	779	1065	0	Histone-lysine N-methyltransferase EZH2 [Zootermopsis nevadensis]
evm.model.Contig498.14#Contig498#273821#275821	14	707	691	0	THO complex subunit 5-like protein [Zootermopsis nevadensis]
evm.model.Contig432.6_evm.model.Contig432.7	2	1014	1328	0	rap guanine nucleotide exchange factor 4 isoform X1 [Tribolium castaneum]
evm.model.Contig327.4#Contig327#44938#46938	1	355	671	0	phosphoribosyl pyrophosphate synthetase-associated protein 2 [Zootermopsis nevadensis]
evm.model.Contig291.22#Contig291#356515#358515	1	508	795	0	delta-coatomer protein [Riptortus pedestris]
evm.model.Contig264.21#Contig264#520783#522783	1	423	662	0	CLP1-like protein [Zootermopsis nevadensis]
evm.model.Contig168.41#Contig168#775717#777717	1	515	821	0	U4/U6 small nuclear ribonucleoprotein Prp4 [Zootermopsis nevadensis]
evm.model.Contig123.134#Contig123#2920088#2922088	1	459	762	0	hypothetical protein [Zootermopsis nevadensis]
evm.model.Contig102.32#Contig102#753600#755600	1	1247	1193	0	ankyrin repeat and SAM domain-containing protein 1A-like isoform X2 [Apis mellifera]
evm.model.Contig78.147.1#Contig78#2208907#2210907	8	724	1127	0	ADAM 17-like protease [Zootermopsis nevadensis]
evm.model.Contig49.30#Contig49#984522#986522	1	1095	1752	0	cation-transporting ATPase [Zootermopsis nevadensis]
evm.model.Contig39.6#Contig39#124325#126325	11	446	644	0	mitochondrial enolase superfamily member 1 [Zootermopsis nevadensis]
evm.model.Contig0.130.1#Contig0#2177241#2179241	48	1107	1254	0	anoctamin-8-like isoform X4 [Bombus impatiens]
evm.model.Contig0.131#Contig0#2175909#2177909	56	415	569	0	ubiquitin carrier protein E2 28 [Zootermopsis nevadensis]
evm.model.Contig0.367#Contig0#7656756#7658756	1	1172	1378	0	structural maintenance of chromosomes protein 2 [Zootermopsis nevadensis]
evm.model.Contig14.153#Contig14#3191013#3193013	1	429	788	0	AT-rich interactive domain-containing protein 4B [Zootermopsis nevadensis]
evm.model.Contig201.21#Contig201#501411#503411	1	848	1052	0	protein kibra isoform X1 [Acyrthosiphon pisum]
evm.model.Contig215.15_evm.model.Contig215.16#	217	759	748	0	adenylate cyclase type 8 isoform X4 [Acyrthosiphon pisum]

evm.model.Contig224.187#Contig224#3147351#3149351	48	630	684	0	glycosylphosphatidylinositol anchor attachment 1 protein [Zootermopsis nevadensis]
evm.model.Contig285.23#Contig285#253881#255881	37	733	1120	0	glycyl-tRNA synthetase [Zootermopsis nevadensis]
evm.model.Contig39.35#Contig39#865498#867498	1	2261	2755	0	DNA polymerase epsilon catalytic subunit A [Athalia rosae]
evm.model.Contig392.5#Contig392#181940#183940	1569	2001	616	0	methylcytosine dioxygenase TET2 isoform X1 [Nasonia vitripennis]
evm.model.Contig45.85#Contig45#2654834#2656834	50	437	742	0	splicing factor u2af large subunit [Riptortus pedestris]
evm.model.Contig485.5#Contig485#235252#237252	1	670	728	0	Ras and EF-hand domain-containing protein-like isoform X1 [Diaphorina citri]
evm.model.Contig64.45#Contig64#953565#955565	1	313	630	0	neuropeptide GPCR A27 [Nilaparvata lugens]
evm.model.Contig685.8#Contig685#143590#145590	14	447	640	0	Dolichyl-diphosphooligosaccharide-protein glycosyltransferase 48 kDa subunit [Zootermopsis nevadensis]
evm.model.Contig692.11#Contig692#287911#289911	22	676	707	0	peroxisomal targeting signal 1 receptor [Zootermopsis nevadensis]
evm.model.Contig85.19#Contig85#425125#427125	23	744	1114	0	elongation factor G 1 [Culex quinquefasciatus]
evm.model.Contig88.3#Contig88#84099#86099	9	1150	1232	0	ribosome biogenesis protein BMS1-like protein [Camponotus floridanus]
evm.model.Contig98.80#Contig98#1858347#1860347	1	883	1147	0	exocyst complex component 1 [Zootermopsis nevadensis]
evm.model.Contig98.31#Contig98#525790#527790	4184	4783	1040	0	E3 ubiquitin-protein ligase HERC2 [Zootermopsis nevadensis]
evm.model.Contig685.7#Contig685#144832#146832	1	381	515	4.00E-178	tetratricopeptide repeat protein 17 [Zootermopsis nevadensis]
evm.model.Contig328.7#Contig328#157392#159392	3	1000	530	2.00E-167	WD repeat-containing protein 6 isoform X1 [Orussus abietinus]
evm.model.Contig248.62#Contig248#1471177#1473177	1	319	475	6.00E-165	WD repeat-containing protein 61 [Zootermopsis nevadensis]
evm.model.Contig21.155#Contig21#3282159#3284159	1	471	478	3.00E-162	RNA-binding protein Musashi homolog 2-like [Diaphorina citri]
evm.model.Contig421.4#Contig421#126965#128965	304	823	494	5.00E-162	centaurin-beta [Pediculus humanus corporis]
evm.model.Contig788.2#Contig788#42744#44744	1	237	463	1.00E-159	sugar transporter [Nilaparvata lugens]
evm.model.Contig283.25#Contig283#983952#985952	1	262	460	2.00E-159	zinc finger protein 330 homolog [Athalia rosae]
evm.model.Contig1069.2#Contig1069#96821#98821	312	653	479	3.00E-159	beta-glucuronidase isoform X1 [Acyrthosiphon pisum]
evm.model.Contig123.24#Contig123#455763#457763	3	231	448	6.00E-157	Proteasome subunit alpha type-7-like [Zootermopsis nevadensis]
evm.model.Contig1140.1#Contig1140#3897#5897	1	303	449	2.00E-152	cytochrome P450 [Laodelphax striatella]

evm.model.Contig2568.1#Contig2568#0#807	8	395	446	4.00E-151	ceramide glucosyltransferase [Monomorium pharaonis]
evm.model.Contig157.66#Contig157#1450545#1452545	5	454	459	5.00E-151	meprin A subunit beta-like [Diaphorina citri]
evm.model.Contig49.31#Contig49#982792#984792	8	395	444	1.00E-150	ceramide glucosyltransferase [Monomorium pharaonis]
evm.model.Contig123.108#Contig123#2330182	1	406	444	3.00E-145	far upstream element-binding protein 3 [Bombus terrestris]
evm.model.Contig39.80#Contig39#1977409#1979409	1	260	419	1.00E-144	trypsin-23 [Nilaparvata lugens]
evm.model.Contig224.68#Contig224#1131256#1133256	3	337	423	2.00E-143	ribosome biogenesis protein BRX1 homolog [Athalia rosae]
evm.model.Contig175.5#Contig175#90755#92755	428	1063	478	4.00E-143	hypothetical protein L798_05958 [Zootermopsis nevadensis]
evm.model.Contig88.30#Contig88#625017#627017	4	313	425	4.00E-143	Homeobox protein prophet of Pit-1 [Camponotus floridanus]
evm.model.Contig79.44_evm.model.Contig79.45	65	539	433	6.00E-143	alanine aminotransferase 1 [Sogatella furcifera]
evm.model.Contig1267.1#Contig1267#18092#20092	1705	1958	440	3.00E-137	uncharacterized protein [Microplitis demolitor]
evm.model.Contig25.137#Contig25#2493744#2495744	435	1405	424	2.00E-127	Nuclear pore complex protein Nup160-like protein [Zootermopsis nevadensis]
evm.model.Contig25.174#Contig25#3156590#3158590	1	322	372	6.00E-123	Ataxin-3 [Zootermopsis nevadensis]
evm.model.Contig528.5#Contig528#166045#168045	166	423	382	2.00E-121	serotonin 5-HT1 receptor [Periplaneta americana]
evm.model.Contig51.11#Contig51#438698#440698	43	219	353	6.00E-121	phosphoserine phosphatase, partial [Nilaparvata lugens]
evm.model.Contig13.198#Contig13#3693922#3695922	1	347	363	1.00E-119	longitudinals lacking protein, isoforms H/M/V-like [Acyrthosiphon pisum]
evm.model.Contig1423.2#Contig1423#44178#46178	504	793	370	8.00E-118	integrin beta-PS [Diaphorina citri]
evm.model.Contig1245.4_evm.model.Contig1245.5	554	958	378	3.00E-111	ubiquitin carboxyl-terminal hydrolase 8 [Zootermopsis nevadensis]
evm.model.Contig64.65#Contig64#1408536#1410536	104	500	359	7.00E-109	hypothetical protein [Dendroctonus ponderosae]
evm.model.Contig92.59#Contig92#949829#951829	31	401	338	1.00E-105	growth/differentiation factor 11 [Megachile rotundata]
evm.model.Contig118.24#Contig118#636225#638225	9	207	314	3.00E-105	transmembrane emp24 domain-containing protein 3 [Zootermopsis nevadensis]
evm.model.Contig139.19#Contig139#322837#324837	18	301	320	3.00E-104	easter-2 [Nilaparvata lugens]
evm.model.Contig1204.3#Contig1204#91926#93926	1	153	306	1.00E-102	putative pyrroline-5-carboxylate reductase 2 [Laodelphax striatella]
evm.model.Contig161.24#Contig161#417665#419665	12	416	311	1.00E-97	hypothetical protein [Lucilia cuprina]
evm.model.Contig0.218#Contig0#3999913#4001913	12	410	302	5.00E-95	forkhead box protein D3 [Pediculus humanus corporis]
evm.model.Contig407.17#Contig407#297620#299620	1	299	288	3.00E-92	polyglutamine-binding protein 1 [Zootermopsis nevadensis]

evm.model.Contig54.37#Contig54#828355#830355	1	152	266	1.00E-87	RNA-binding protein 8A-like [Coptotermes formosanus]
evm.model.Contig3269.4_evm.model.Contig3269.5	34	662	293	2.00E-85	ribosomal RNA processing protein 1 homolog isoform X2 [Megachile rotundata]
evm.model.Contig219.68#Contig219#1319915#1321915	1	138	251	8.00E-83	trafficking protein particle complex subunit 2 [Zootermopsis nevadensis]
evm.model.Contig111.13#Contig111#213581#215581	1	226	258	6.00E-81	S-formylglutathione hydrolase [Crassostrea gigas]
evm.model.Contig276.5#Contig276#109526#111526	1	412	265	5.00E-80	transcription factor deformed [Tribolium castaneum]
evm.model.Contig166.51#Contig166#1259593#1261593	1	311	255	6.00E-78	maternal protein exuperantia [Diaphorina citri]
evm.model.Contig137.40#Contig137#595907#597907	1	177	243	8.00E-78	Fas apoptotic inhibitory molecule [Zootermopsis nevadensis]
evm.model.Contig479.5#Contig479#70004#72004	623	926	275	8.00E-74	ankyrin repeat and LEM domain-containing protein 1 [Danio rerio]
evm.model.Contig109.47#Contig109#955497#957497	25	613	253	3.00E-71	bifunctional ATP-dependent dihydroxyacetone kinase/FAD-AMP lyase-like isoform X2 [Orussus abietinus]
evm.model.Contig0.14#Contig0#286990#288990	1	167	216	5.00E-68	RhoA activator C11orf59-like protein [Zootermopsis nevadensis]
evm.model.Contig170.52#Contig170#1156300#1158300	3	118	189	3.00E-58	zinc finger protein 593 homolog [Bombus impatiens]
evm.model.Contig36.32#Contig36#621292#623292	1	182	197	1.00E-56	RNA-binding protein 48 [Athalia rosae]
evm.model.Contig157.73#Contig157#1616449#1618449	140	500	218	1.00E-54	snRNA-activating protein complex subunit 4 [Zootermopsis nevadensis]
evm.model.Contig3873.1#Contig3873#3029#5029	1	90	176	2.00E-52	proteasome 28kD subunit 1 [Riptortus pedestris]
evm.model.Contig261.20#Contig261#454455#456455	1	123	169	4.00E-51	protein LAMTOR3-A [Fopius arisanus]
evm.model.Contig18968.1#Contig18968#6484#8484	1	302	186	2.00E-50	WD repeat-containing protein 6, partial [Nasonia vitripennis]
evm.model.Contig0.46#Contig0#680634#682634	29	202	177	4.00E-47	zinc finger protein 34-like isoform X1 [Astyanax mexicanus]
evm.model.Contig36.5#Contig36#129660#131660	1	116	153	5.00E-45	protein C10 [Bombus terrestris]
evm.model.Contig682.3#Contig682#4122#6122	421	505	167	7.00E-45	kazrin-A, partial [Zootermopsis nevadensis]
evm.model.Contig3975.1#Contig3975#417#2417	46	197	167	1.00E-44	mitogen-activated protein kinase kinase kinase 12 isoform X2 [Athalia rosae]
evm.model.Contig598.5#Contig598#113808#115808	1	115	151	3.00E-44	allatotropin [Nilaparvata lugens]
evm.model.Contig974.7#Contig974#202101#204101	13	284	176	3.00E-43	PHD finger protein 12 [Tribolium castaneum]
evm.model.Contig175.4#Contig175#90279#92279	3	678	171	7.00E-42	protein PAT1 homolog 1 isoform X1 [Megachile rotundata]
evm.model.Contig100.187#Contig100#4194046	1	203	145	2.00E-39	hypothetical protein [Pediculus humanus corporis]
evm.model.Contig129.2#Contig129#11760#13760	40	130	148	3.00E-39	polypyrimidine tract-binding protein 1 isoform X4 [Pogonomyrmex barbatus]

evm.model.Contig1012.1#Contig1012#24937#26937	1476	1755	151	2.00E-37	Cell division cycle 2-like protein kinase [Zootermopsis nevadensis]
evm.model.Contig0.13#Contig0#287507#289507	1	313	154	4.00E-37	hypothetical protein [Zootermopsis nevadensis]
evm.model.Contig7160.1#Contig7160#0#1084	118	187	137	2.00E-36	hypothetical protein [Zootermopsis nevadensis]
evm.model.Contig10858.1#Contig10858#21584#23584	426	563	139	1.00E-35	solute carrier organic anion transporter family member 4C1-like isoform X2 [Acyrthosiphon pisum]
evm.model.Contig54.35#Contig54#828341#830341	115	425	140	2.00E-33	kinesin-like protein KIF3A [Sorex araneus]
evm.model.Contig78.108#Contig78#1823701#1825701	1	105	124	7.00E-33	chromatin accessibility complex protein 1 [Nasonia vitripennis]
evm.model.Contig1154.2#Contig1154#6110#8110	150	424	131	1.00E-30	hypothetical protein [Zootermopsis nevadensis]
evm.model.Contig15.54#Contig15#1413523#1415523	735	833	117	4.00E-28	DNA replication licensing factor MCM8 [Zootermopsis nevadensis]
evm.model.Contig665.4#Contig665#65844#67844	92	395	122	6.00E-28	mRNA-decapping enzyme 1B isoform X2 [Bombyx mori]
evm.model.Contig784.3#Contig784#115321#117321	44	157	107	1.00E-26	ACYPI006691 [Acyrthosiphon pisum]
evm.model.Contig931.4#Contig931#55531#57531	27	323	119	1.00E-25	hypothetical protein [Zootermopsis nevadensis]
evm.model.Contig760.14#Contig760#250267#252267	1	146	115	2.00E-24	uncharacterized protein [Athalia rosae]
evm.model.Contig170.51#Contig170#1157311#1159311	2	256	103	2.00E-22	uncharacterized protein [Plutella xylostella]
evm.model.Contig554.4#Contig554#50028#52028	6978	7044	100	6.00E-22	twitchin isoform X22 [Monomorium pharaonis]
evm.model.Contig3197.1#Contig3197#4167#6167	5	118	99	1.00E-21	uncharacterized protein [Pogonomyrmex barbatus]
evm.model.Contig45.94#Contig45#2847889#2849889	5	66	89.4	3.00E-21	26S proteasome complex subunit DSS1-like [Tupaia chinensis]
evm.model.Contig1154.1#Contig1154#6497#8497	166	297	98.2	3.00E-20	hypothetical protein [Dendroctonus ponderosae]
evm.model.Contig100.155#Contig100#3608233	17	102	86.7	1.00E-18	predicted protein [Nematostella vectensis]
evm.model.Contig58.115#Contig58#1978658#1980658	12	256	92.8	4.00E-18	uncharacterized protein [Plutella xylostella]
evm.model.Contig78.117#Contig78#1986267#1988267	181	389	88.2	7.00E-16	protein spindly [Zootermopsis nevadensis]
evm.model.Contig1660.2#Contig1660#10944#12291	389	510	80.1	6.00E-14	laminin subunit alpha-1-like [Diaphorina citri]
evm.model.Contig39.11#Contig39#417698#419698	302	354	77.4	4.00E-13	uncharacterized protein LOC105389939 isoform X1 [Plutella xylostella]
evm.model.Contig1304.1#Contig1304#10724#12724	90	166	72.4	3.00E-12	head-specific guanylate cyclase [Tribolium castaneum]
evm.model.Contig39.20#Contig39#548761#550761	36	250	73.6	4.00E-11	hypothetical protein L798_06445 [Zootermopsis nevadensis]
evm.model.Contig78.116.1#Contig78#1984432#1986432	396	980	74.3	4.00E-10	leucine-rich repeat and coiled-coil domain-containing protein 1-like isoform X2 [Crassostrea gigas]
evm.model.Contig665.5#Contig665#64306#66306	1	68	59.3	2.00E-09	AGAP012215-PA [Anopheles gambiae str. PEST]
evm.model.Contig760.16#Contig760#258740#260740	66	176	61.6	1.00E-07	uncharacterized protein LOC103311965 [Acyrthosiphon pisum]

evm.model.Contig3197.2#Contig3197#4843#6843	150	205	55.1	8.00E-07	28S ribosomal protein S30, mitochondrial [Zootermopsis nevadensis]
evm.model.Contig36.77#Contig36#1269006#1271006	149	200	53.9	2.00E-06	predicted acetyltransferase [Aedes aegypti]
evm.model.Contig17.74#Contig17#1751845#1753845	91	136	56.6	3.00E-06	hypothetical protein L798_00184 [Zootermopsis nevadensis]
evm.model.Contig544.2#Contig544#67639#69639	21	105	47.8	6.00E-05	MGC81028 protein [Xenopus laevis]

Table S3. Primers used in this study.

Primer name	Sequence (5' to 3')
**Primer used for qPCR**	
RNA3-IR-F	TCATGACCCAAAAAACTGCACACCACTG
RNA3-IR-R	TTGGCCAATCATGGCTTTAGGCAAAAG
NP-F	GATGAAGTACACAACTGGTC
NP-R	AGTGCTGATCGTATTGACAGA
EF2-F	GTCTCCACGGATGGGCTTT
EF2-R	ATCTTGAATTTCTCGGCATACATTT
importin α1-F	AAGAGTTGGAGAGCAAGAAGCA
importin α1-R	CGGTAGTCCTCGTCCTCTGT
importin α2-F	CAGTGATGCTCAGACGGATTCG
importin α2-R	GCGGTGACATTGGACACAGTC
importin α3-F	GCGAAGGAGGCGGAATGAA
importin α3-R	CGATGCGTCCATAACCAACTG
YY1-F	GTGTATGACCAGATCCCAGTACCG
YY1-R	GTTCCTGAAGCCCACATTGTCA
FAIM-F	GAGTGATATGGTTCACAGG
FAIM-R	TTGAACATCCACTCTTTCC
P450-F	GGGAGGCAGTGAGACCACAGC
P450-R	GGCGAAGTCAAGTTCCTGATCG
E2-F	TCGAGGAACCCGTTTGTGCG
E2-R	GCCCGAATCTCCATCGTCAG
UBTH8-F	GAAGGTGGATTCAGGGAGTGGC
UBTH8-R	CAGATGGAGCAGGCGCTGTG
HERC2-F	GGAGCACAGACAAGCCGATGC
HERC2-R	CGACCACGGGACAGAGCAGG
PTSR-F	CCGGATCAACTGGTGCAGC
PTSR-R	TCTTCATTGGCTTCGGCATC
FAIM-promoter-F	GAAGTTGTTGCTCAAAGAGAACTGC
FAIM-promoter-R	TCTGGGTTAGACTATCGTCAGTTTTC
P450-promoter-F	AGAGGTAATTTGACGGTAGGTGAC
P450-promoter-R	CATGTATAACATGACCGCCTTCC
E2-promoter-F	GGGATTTCGTTACTGCGGC
E2-promoter-R	TGCACCATGTATACACCCGC
UBTH8-promoter-F	AAGCCGCCGTCGAAGAAG
UBTH8-promoter-R	TTGGATTGTGAAAGGGCTTG
HERC2-promoter-F	GGGCGACAGATAACAGTTCTC
HERC2-promoter-R	CATAGCTGAAATCAGCAGCAC
PTSR-promoter-F	GACCAACACGAACACAAACG
PTSR-promoter-R	CTTTACCAAGAAAGCCACTCC
**Primers used for RT-PCR**	
RNA1-5'NTR-F	ATCGGCATAGACAATGAACTGTTG
RNA1-5'NTR-R	ATCGGCATAGACAATGAACTGTTG
RNA2-IR-F	ATCGGCATAGACAATGAACTGTTG
RNA2-IR-R	ATCGGCATAGACAATGAACTGTTG
RNA3-IR-F	ATCGGCATAGACAATGAACTGTTG
RNA3-IR-R	ATCGGCATAGACAATGAACTGTTG
RNA4-IR-F	ATCGGCATAGACAATGAACTGTTG
RNA4-IR-R	ATCGGCATAGACAATGAACTGTTG
**Primers used for RNAi**	
importin α1-dsRNA-F	GAACAGCGGAGGCGGAATA
importin α1-dsRNA-R	GAGCAGTTGAGCACCACTTG
importin α2-dsRNA-F	AGGAATGATGAGCAGCGATGA
importin α2-dsRNA-R	CGGAAGCGGAGACTTGTGT
importin α3-dsRNA-F	CCTCTCCTCATCGCACCAA

importin α3-dsRNA-R	GCCACCGCATTCCTCAATC
YY1-dsRNA-F	TGAGGGCAGTAACCCAGAA
YY1-dsRNA-R	CACATCAGCCACTTCCACC
GFP-dsRNA-F	CACAAGTTCAGCGTGTCCG
GFP-dsRNA-R	GTTCACCTTGATGCCGTTC
FAIM-dsRNA-F	GGCACTACTACAGGCAAGAG
FAIM-dsRNA-R	GTACATCAACAAATAGTTCCTCT
importin α1-T7-F	TAATACGACTCACTATAGGGAACAGCGGAGGCGGAATA
importin α1-T7-R	TAATACGACTCACTATAGGGAGCAGTTGAGCACCACTTG
importin α2-T7-F	TAATACGACTCACTATAGGAGGAATGATGAGCAGCGATGA
importin α2-T7-R	TAATACGACTCACTATAGGCGGAAGCGGAGACTTGTGT
importin α3-T7-F	TAATACGACTCACTATAGGCCTCTCCTCATCGCACCAA
importin α3-T7-R	TAATACGACTCACTATAGGGCCACCGCATTCCTCAATC
YY1-T7-F	TAATACGACTCACTATAGGTGAGGGCAGTAACCCAGAA
YY1-T7-R	TAATACGACTCACTATAGGCACATCAGCCACTTCCACC
GFP-T7-F	TAATACGACTCACTATAGGCACAAGTTCAGCGTGTCCG
GFP-T7-R	TAATACGACTCACTATAGGGTTCACCTTGATGCCGTTC
FAIM-T7-F	TAATACGACTCACTATAGGGGCACTACTACAGGCAAGAG
FAIM-T7-R	TAATACGACTCACTATAGGGTACATCAACAAATAGTTCCTCT
**Primers used for protein expression**	
importin α1-antigen-F	AGGAGATATACCATGGGCTGCTGGACCATTTCCAACAT
importin α1-antigen-R	GACGGAGCTCGAATTCTTAGTGGTGGTGGTGGTGGTGGAACT CGAATCCGCCCAT
importin α2-antigen-F	AGGAGATATACCATGGGCGTTATCGAAGCTGGAGTTCT
importin α2-antigen-R	GACGGAGCTCGAATTCTTAGTGGTGGTGGTGGTGGTGAAAAG AGAATCCTCCGTCTG
importin α3-antigen-F	AGGAGATATACCATGGGCACAGCTGGAAACCAACACC
importin α3-antigen-R	GACGGAGCTCGAATTCTTAGTGGTGGTGGTGGTGGTGAAACT TGAAGCCTTCGCTAG
importin α1-his-F	AGGAGATATACCATGGGCATGTCCGGCCATAAGTATCG
importin α1-his-R	GACGGAGCTCGAATTCTTAGTGGTGGTGGTGGTGGTGGAACT CGAATCCGCCCAT
importin α2-his-F	AGGAGATATACCATGGGCATGAGACGACGAAGAAATG
importin α2-his-R	GACGGAGCTCGAATTCTTAGTGGTGGTGGTGGTGGTGAAAAG AGAATCCTCCGTC
importin α3-his-F	AGGAGATATACCATGGGCATGGCGTCCGATCAAATA
importin α3-his-R	GACGGAGCTCGAATTCTTAGTGGTGGTGGTGGTGGTGAAACT TGAAGCCTTCGCTA
YY1-his-F	GGAATTCCATATGATGGCGTCCG
YY1-his-R	GAATTCATCGGCATAGACAATGAACTGTTG
NP-cDNA-F	ATGGGTACCAACAAGCCAGCC
NP-cDNA-R	CTAGTCATCTGCACCTTCTGCC
NP-Flag-F	GATTACAAGGATGACGACGATAAGATGGGTACCAAC
NP-Flag-R	CTAGTCATCTGCACCTTCTGCCTCATCC
NP-Flag-N-F	GATTACAAGGATGACGACGATAAGATGGGTACCA
NP-Flag-N-R	TTGTGGAACATAGTCCCACAGTAAGTTG
NP-Flag-C-F	GATTACAAGGATGACGACGATAAGTATATCAAAC
NP-Flag-C-R	CTAGTCATCTGCACCTTCTGCCTC
pAc5.1B-NP-F	AATTCTGCAGATATCATGGGTACCAACAAGCCAGCCAC
pAc5.1B-NP-R	ACCTTCGAACCGCGGTTACTTGTCATCGTCGTCCTTGTAGTCG TCATCTGCACCTTCTGCCTC
GFP-F	GGTACCATGAGTAAAGGAGAAGAAC
GFP-R	TTTGTATAGTTCATCCATGCCATG
GFP-linker-R	GCTGCCGCTGCCGCTGCCTTTGTATAGTTCATC
NP-NLS-BCD-linker-F	GGCAGCGGCAGCGGCAGCGAGGGTAAGAAG
NP-NLS-BCD-F	GAGGGTAAGAAGAAGGAGC

NP-NLS-BCD-R	CTAGTCATCTGCACCTTCTGCC
**Primers used for probe synthesis of RSV RNA3**	
SP6-RNA3-F	GAATTGATTTAGGTGACACTATAGCTCTAGTTGGTAGAGAAA
T7-RNA3-R	GAATTGTAATACGACTCACTATAGGGTGAAACAGCTCCTTAC

F, forward primer; R, reverse primer.
